# Lactoferrin Protects against Methamphetamine Toxicity by Modulating Autophagy and Mitochondrial Status

**DOI:** 10.3390/nu13103356

**Published:** 2021-09-25

**Authors:** Larisa Ryskalin, Francesca Biagioni, Carla L. Busceti, Maico Polzella, Paola Lenzi, Alessandro Frati, Michela Ferrucci, Francesco Fornai

**Affiliations:** 1 Department of Translational Research and New Technologies in Medicine and Surgery, University of Pisa, Via Roma 55, 56126 Pisa, Italy; larisa.ryskalin@unipi.it (L.R.); paola.lenzi@unipi.it (P.L.); michela.ferrucci@unipi.it (M.F.); 2 Istituto di Ricovero e Cura a Carattere Scientifico (I.R.C.C.S.) Neuromed, Via Atinense 18, 86077 Pozzilli, Italy; francesca.biagioni@neuromed.it (F.B.); carla.busceti@neuromed.it (C.L.B.); alessandro.frati@uniroma1.it (A.F.); 3 Aliveda Laboratories, Viale Karol Wojtyla, 19, 56042 Crespina Lorenzana, Italy; maico@aliveda.com; 4 Neurosurgery Division, Human Neurosciences Department, Sapienza University, 00135 Rome, Italy

**Keywords:** autophagy, lactoferrin, methamphetamine, cell death, light microscopy

## Abstract

Lactoferrin (LF) was used at first as a vehicle to deliver non-soluble active compounds to the body, including the central nervous system (CNS). Nonetheless, it soon became evident that, apart from acting as a vehicle, LF itself owns active effects in the CNS. In the present study, the effects of LF are assessed both in baseline conditions, as well as to counteract methamphetamine (METH)-induced neurodegeneration by assessing cell viability, cell phenotype, mitochondrial status, and specific autophagy steps. In detail, cell integrity in baseline conditions and following METH administration was carried out by using H&E staining, Trypan blue, Fluoro Jade B, and WST-1. Western blot and immuno-fluorescence were used to assess the expression of the neurofilament marker βIII-tubulin. Mitochondria were stained using Mito Tracker Red and Green and were further detailed and quantified by using transmission electron microscopy. Autophagy markers were analyzed through immuno-fluorescence and electron microscopy. LF counteracts METH-induced degeneration. In detail, LF significantly attenuates the amount of cell loss and mitochondrial alterations produced by METH; and mitigates the dissipation of autophagy-related proteins from the autophagy compartment, which is massively induced by METH. These findings indicate a protective role of LF in the molecular mechanisms of neurodegeneration.

## 1. Introduction

Lactoferrin (LF) is an 80 kDa iron-binding multifunctional glycoprotein belonging to the transferrin family [1]. It is highly conserved among mammalian species [2,3,4,5,6]. LF occurs within mucosal secretions [7,8], including colostrum and milk [9], and within neutrophil granules [10]. LF was used at first as a vehicle to deliver non-soluble active compounds to the body, including the central nervous system (CNS) [11,12,13,14,15,16,17,18]. It soon became evident that, apart from acting as a vehicle, LF itself owns active effects in the CNS. In fact, strong protective effects are hypothesized to take place in a variety of neuropathological conditions, including degenerative disorders [19].

Within the CNS, LF occurs in various cells [20,21]. For instance, microglia contain LF [22,23], which is also present within neurons both in baseline conditions and specific disorders [20]. In fact, the overall production of LF increases within CNS in degenerative dementia and Parkinsonism [20,24,25,26]. In Alzheimer’s disease (AD), LF expression greatly increases within cortical neurons [20,25], and it is present within amyloid deposits and amyloid angiopathy in AD models [27]. In Parkinson’s disease (PD), LF increases within surviving dopamine (DA)-containing neurons within substantia nigra pars compacta (SNpc) [26]. Experimental models of Parkinsonism recapitulate PD-induced increase in LF within midbrain neurons [28,29].

It is remarkable that, LF appears to protect neurons from the very same disorders featuring endogenous overexpression of LF. This is the case of dementia [30,31,32] and experimental Parkinsonism [33,34,35,36,37,38].

LF owns intrinsic antioxidant properties [14,36,37,39], which are partly related to the modulation of iron metabolism [33,36,37]. However, most neuroprotection by LF does not depend on iron metabolism.

The protective role of LF is likely to recruit specific intra-cellular pathways. In fact, within DA, LF neurons trigger phospho-AKT-mediated calcium shuttling from endoplasmic reticulum to mitochondria [34]. In recent years, evidence indicates that LF promotes autophagy [14,40,41,42]. In turn, autophagy modulation is relevant in the course of a variety of degenerative conditions [43,44,45]. At present, the measurement of LF-induced autophagy is not provided, and that of subcellular effects of LF on autophagy-dependent structures (autophagy vacuoles and mitochondria) is missing.

Therefore, the present study directly analyzes the involvement of autophagy in mediating LF-induced neuroprotection. In detail, the potential neuroprotection of LF against methamphetamine (METH) toxicity was investigated. In fact, the widely abused psychostimulant METH specifically alters the autophagy machinery [46], as well as the mitochondrial status [47,48]. In fact, the molecular mechanisms of METH involve a number of steps. In detail, METH blocks and reverts the dopamine (DA) transporter (DAT) [49,50]; it inhibits and displaces the vesicular monoamine transporter type-2 (VMAT-2) [51,52,53] and disrupts the vesicular proton gradient [54,55,56]. This produces a massive DA efflux from the vesicles towards the cytosol [57,58,59]. Such a high amount of cytosol DA cannot be properly metabolized by monoamine oxidase (MAO), also due to a competitive inhibition by METH of MAO-A [60,61]. As a consequence, freely non-metabolized DA auto-oxidizes, which triggers a powerful oxidative stress, which, in turn, generates highly reactive DA by-products [62,63,64,65]. These include 3,4-dihydroxyphenylacetaldehyde (DOPALD) and its self-oxidation by-product, DA quinone. Highly reactive oxidative species (ROS) produced by METH alter protein structures to produce protein misfolding and mitochondrial impairment [62,63,64,65,66,67]. Recent studies indicate that these alterations are relevant for METH-induced neurodegeneration [46,68]. The usefulness of METH also relies on its ability to mimic a number of degenerative disorders [69]. In fact, it is known that METH abuse fosters Parkinsonism in various animal species, including humans [70,71], and produces cognitive alterations [72,73,74,75]. Thus, the chance that LF may protect against METH toxicity deserves specific investigations in an effort to mitigate the neurotoxic effects of a widely abused psychostimulant and to disclose a potential compound acting as a disease-modifier in the course of common mechanisms of neurodegeneration. METH was administered in vitro to DA-containing PC12 cells since this model is well characterized, both concerning specific cell pathology and involvement of the autophagy machinery [46,47,54,66,68,76]. The low experimental variables, inherent to such a controlled experimental setting, represent an add on to identify the molecular mechanisms responsible for a potential protection induced by LF. In addition, such a model allows oneto rule out anti-inflammatory and immunomodulatory effects of LF to directly assess the impact of LF on autonomous cell protective pathways, independently of glia, inflammatory cells, and brain vessels [35,77,78].

In the present study, the bovine isoform of LF (bLF, from now on simply LF) was selected due to high sequence homology with human LF [79] and a large availability and safety as certified by Food and Drug Administration (FDA) [80,81]. In this model, the effects of LF were assessed both in baseline conditions and in the presence of METH, concerning alterations of cell viability, cell phenotype, mitochondrial status, and specific autophagy steps. To summarize, the present study investigates the following: (i) whether LF is beneficial or detrimental for cell viability; (ii) whether LF neuroprotection following METH occurs; (iii) whether this occurs as a cell autonomous activity, independently by concomitant mechanisms; (iv) and whether this relies on specific subcellular targets such as the autophagy machinery and mitochondria.

## 2. Materials and Methods

### 2.1. Experimental Design

Dose–response and time-course of PC12 cell survival produced by various doses of LF (from 0.16 μM to 320 μM) during various times of exposure (from 24 h up to 168 h). This allows one to select the optimal doses of LF and the best time of exposure to assess the following:

(i) The occurrence of phenotypic changes and altered cell viability produced by LF alone.
(ii) The potential protective effects of LF on METH-induced toxicity. The METH dose used in the present study was 100 μM, which has been recently demonstrated [48] to produce a moderate toxicity in PC12 cells after 72 h of exposure.
(iii) The effects of LF on METH-induced mitochondrial alterations.
(iv) The involvement of specific autophagy steps in the protective effects of LF against METH-induced toxicity.

In order to assess whether the effects of LF were specific for METH-induced neurodegeneration of DA-containing cells, we used a negative control. The negative control consisted in a non-neuronal cell line lacking DA (which is key to mediate METH-induced damage). To this aim, we used glioblastoma (GBM)-derived U87MG cells to validate the specificity of the neuroprotection exerted by LF. 

### 2.2. Cell Cultures

Experiments were carried out in the rat pheochromocytoma PC12 cell line obtained from a cell bank (IRCCS San Martino Institute, Genova, Italy). PC12 cells were grown in 75 cm^2^ tissue culture flask containing RPMI 1640 medium (Sigma-Aldrich, St. Louis, MO, USA) supplemented with 10% heat-inactivated horse serum (HS; Sigma-Aldrich), 5% fetal bovine serum (FBS; Sigma-Aldrich), penicillin (50 IU/mL), and streptomycin (50 mg/mL; Sigma-Aldrich). Cells were kept under standard culture conditions at 37 °C in a humidified atmosphere containing 5% CO_2_. Cells were in log phase of growth, at approximately 70% confluence [82,83], when used for the present experiments.

For light microscopy experiments, 5 × 10^4^ PC12 cells were seeded on polylysine cover slips, which were placed in 24-well plates in a final volume of 1 mL/well.

For Western blotting assay, 5 × 10^5^ cells were seeded in six-well plates in a final volume of 2 mL/well.

For transmission electron microscopy (TEM) experiments, 10^6^ cells were seeded in culture dishes in a final volume of 5 mL.

Uppsala derived human cell line (U87MG) obtained from Cell Bank (IRCCS San Martino-Institute, Genova, Italy) were used in additional experiments as negative controls. U87MG cells were maintained in DMEM growth medium (Sigma-Aldrich, Saint Louis, MO, USA) containing 10% Fetal Bovine Serum (FBS, Sigma-Aldrich), 1% of MEM Non-Essential Amino-Acid (MEM-NEAA, Sigma-Aldrich), penicillin, and streptomycin (50 IU/mL and 100 μg, respectively, Sigma-Aldrich) and kept at 37 °C in a humidified atmosphere containing 5% CO_2_.

For cell count and immuno-fluorescence experiments, 5 × 10^4^ U87MG cells were seeded on cover slips, which were put down within 24-well plates in a final volume of 1 mL/well.

For Western blot and TEM, 1 × 10^6^ U87MG cells were seeded in 6-well plates, in a final volume of 2 mL/well.

### 2.3. Cell Treatments

Stock solutions of LF and METH were prepared to carry out cell treatments. A stock solution of LF (Sigma-Aldrich), 1.28 mM, was obtained by dissolving 4 mg of LF powder in 1 mL of basal medium, while a stock solution of METH (kindly gifted by Forensic Medicine, University of Pisa), 10 mM, was prepared by dissolving 2.3 mg of METH directly in 1 mL of basal medium. Appropriate aliquots of LF and METH stock solutions were diluted within the cell culture medium to obtain the final treatment solutions.

In detail, LF was administered at doses of 0.16, 1.6, 16, 160, and 320 μM for 24, 48, 72, and 168 h to select the safe LF doses. The following experiments were carried out by administering LF at doses of 1.6 and 16 μM for 72 h, alone or in combination with METH at the dose of 100 μM. When LF and METH were given in combination, LF was administered 2 h before METH.

In combined LF + METH treatments, LF was added to the culture medium at concentrations of 1.6 and 16 μM 2 h before METH administration at the dose of 100 μM. After 72 h, cells were collected and analyzed according to the following procedures: H&E, WST-1, TB, and FJB; measurement of cell branches and βIII-tubulin expression by immuno-fluorescence and Western blotting; analysis of mitochondria through MTR-G and MTR-R, along with ultrastructural morphometry at TEM; measurements of specific autophagy proteins by immuno-fluorescence and immunogold-based ultrastructural stoichiometry at TEM.

### 2.4. WST-1 Assay

For WST-1 cell viability assay, PC12 or U87MG cells were seeded at the density of 10^4^ cells/well and placed within 96-well plates in 100 μL of culture medium.

At the end of the treatments, cell viability was assessed using the cell proliferation reagent WST-1 (4-[3-(4-Iodophenyl)-2-(4-nitro-phenyl)-2H-5-tetrazolio]-1,3-benzene sulfonate, Roche Diagnostics GmbH, Mannheim, Germany) according to the manufacturer’s protocol. Briefly, 10% WST-1 reagent was added to each well, and the cells were incubated for 1 h at 37 °C and 5% CO_2_. Cell viability was measured at 450 nm in a microplate reader (BioTek Instruments, Winooski, VT, USA). Data were obtained in three independent experiments and given as the mean percentage ± S.E.M. percentage (assuming control as 100% WST-1).

### 2.5. Trypan Blue Staining

For trypan blue (TB) staining, PC12 or U87MG cells were seeded at a density of 10^4^ cells/well and placed within 24-well plates in 1 mL of culture medium 24 h before treatment. At the end of the treatments, PC12 cells were collected and centrifuged at 800× *g* for 5 min, to obtain a cell pellet. The cell pellet was suspended in the culture medium, and 25 μL of the cell suspension were added to a solution containing 1% TB (62.5 μL, Sigma-Aldrich) and PBS (37.5 μL) for 10 min at room temperature. Cell count was carried out by analyzing 10 μL of this latter solution within a Bürker glass chamber under an Olympus CKX 41 inverted microscope (Olympus Corporation, Tokyo, Japan). Viable and non-viable cells were counted, data are given as the mean percentage ± S.E.M. percentage of TB-positive cells out of total cells. Data were obtained from three independent experiments.

### 2.6. Fluoro Jade B Staining

PC12 or U87MG cells were washed in PBS and fixed with 4% paraformaldehyde for 5 min, incubated with 0.06% potassium permanganate for 10 min at room temperature, and then washed with distilled water. A Fluoro Jade B (FJB, Merck Millipore Billerica, MA, USA) solution was prepared by dissolving 0.01% FJB in acetic acid. Cells were incubated with 0.0004% of this FJB solution for 20 min at room temperature and then cover slipped with mounting medium. FJB-positive cells were analyzed using a Nikon Eclipse 80i light microscope (Nikon, Tokyo, Japan), equipped with a fluorescence lamp and a digital camera connected to the NIS Elements software for image analysis (Nikon, Tokyo, Japan). For each experimental group, the count of FJB-positive cells and the measure of the fluorescence intensity were carried out. In detail, the number of FJB-positive cells was counted at 20× magnification within 5 distinct microscopic fields, where only distinct, not overlapped cells were counted. The intensity of the fluorescent signaling was measured under florescence microscopy in 50 cells/group, using the software Image J. Values are given as the mean number ± S.E.M. of FJB-positive cells and the mean percentage ± S.E.M. percentage of optical density for each experimental group (assuming controls as 100%). The data refer to three independent experiments.

### 2.7. Hematoxylin and Eosin (H&E) Histochemistry

Cells were fixed with 4% paraformaldehyde in PBS for 15 min at room temperature, washed with PBS, and then stained with hematoxylin solution (Sigma-Aldrich). Hematoxylin staining was stopped by washing in running water. Then, cells were stained with eosin solution (Sigma-Aldrich). After repeated washing with distilled water to remove the excess dye, cells were dehydrated in increasing ethylic alcohol solutions, clarified in xylene, and finally covered with DPX mounting medium (Sigma-Aldrich). H&E-stained cells were observed under the Nikon Eclipse 80i light microscope (Nikon). To evaluate cell viability, H&E-stained cells were counted under 20× magnification. Cell counts were carried out within 5 distinct microscopic fields, where only distinct, not overlapped cells were counted. Values are given as the mean percentage ± S.E.M. percentage of cells counted in three independent experiments (assuming control cells as 100%).

Phenotypic changes (i.e., number of cells with branches and branches length) induced by LF were measured in H&E-stained cells at light microscopy (Nikon) at 40× magnification for PC12 cells or 20× magnification for U87MG cells. The number of cells with branches was counted in 100 cells/group in PC12 cells, while the number of branches per cell was counted in 50 cells/group in U87MG cells. Values are given as mean percentage ± S.E.M. percentage or mean number ± S.E.M. The lengths of cell branches were measured in μM by the free software Image J, and values are given as mean ± S.E.M. The data refer to three independent experiments.

### 2.8. Autophagy and Mitochondria Study

The potential involvement of the autophagy pathway in LF-induced protection against METH toxicity was investigated by evaluating the expression of specific autophagy-related proteins by immuno-fluorescence and immunocytochemistry. Double immuno-fluorescence at light microscopy was carried out for the autophagy marker LC3 and the lysosome-related antigens, Cathepsin D and LAMP1, as reported in paragraph s Immunocytochemistry at Light Microscopy. In addition, plain TEM was implemented by post-embedding immunocytochemistry for LC3 and LAMP1

The mitochondria status was analyzed both at light and electron microscopy. Mitochondrial staining of healthy and total mitochondria was assessed by MitoTracker-Red (MTR-R) and MitoTracker-Green (MTG-G) staining, respectively, as reported in Mitochondrial Labelling. At TEM total and altered mitochondria were quantified by ultrastructural morphology, based on criteria which were previously validated [71], and reported in. Transmission Electron Microscopy and. Ultrastructural Morphometry.

### 2.9. Mitochondrial Labelling

To stain mitochondria in living cells, MTR-R (Thermo-Fisher Scientific, Waltham, MA, USA) and MTR-G (Thermo-Fisher Scientific) dyes were used, which allow to reveal healthy (MTR-R) and total (both healthy and unhealthy, MTR-G) cell mitochondria, respectively [84,85,86].

Briefly, 5 × 10^4^ PC12 cells were grown in 24-well plates containing 1 mL/well of culture medium. At the end of each experiment, the medium was removed, and cells were incubated in a solution of MTR-R or MTR-G at 500 nM in a serum free culture medium for 45 min, at 37 °C and 5% CO_2_. At the end of incubation, MTR-R or MTR-G solution was removed and fresh pre-warmed medium was added. Stained cells were analyzed at fluorescence microscopy (Nikon), and the optical density was measured using Image J software (Version 1.8.0_172). Values are given as the mean percentage ± S.E.M. percentage of the optical density measured in 50 cells/group. Data refer to three independent experiments.

### 2.10. Immunocytochemistry at Light Microscopy

PC12 cells were washed in PBS, fixed with 4% paraformaldehyde for 5 min at RT, and permeabilized by Triton X 0.1% (Sigma-Aldrich) for 15 min in PBS. Then, cells were incubated overnight at 4 °C with the primary antibody solution, which was prepared by adding specific concentrations of different primary antibodies to 2% NGS and PBS. Afterwards, cells were incubated for 1 h with the anti-rabbit fluorophore-conjugated secondary antibodies (Alexa 488, Life Technologies, Carlsbad, CA, USA) diluted 1:200 or the anti-mouse fluorophore-conjugated secondary antibodies (Alexa 488 or 546; Life Technologies) diluted 1:200 in PBS at RT. All these steps were carried out within the well plate. After washing in PBS, DAPI (Sigma-Aldrich) 1:1000 was added to visualize cell nuclei; then, slices were gently pulled out to be transferred on a coverslip and mounted with the mounting medium Fluoroshield (Sigma-Aldrich). Cells were observed under fluorescence microscopy (Nikon). In this study, the following primary antibodies were used: rabbit anti-LC3 antibody (Abcam Cambridge, UK) diluted 1:100, mouse anti-βIII-tubulin (Abcam) diluted 1:200, mouse anti-Cathepsin D antibody (Sigma-Aldrich) diluted 1:1000, and mouse anti-LAMP1 antibody (GeneTex, Irvine, CA, USA) diluted 1:100. Immuno-fluorescence for βIII-tubulin was carried also in U87MG cells, following the very same experimental procedure. 

Double immuno-fluorescence was carried out by incubating cells overnight at 4 °C with the primary antibody solution containing both the anti-LC3 and either anti-Cathepsin D or anti-LAMP1 antibodies. Merging of fluorescence was obtained through NIS Elements software (Nikon). All experiments were carried out in triplicate.

### 2.11. Western Blotting

Cells were lysed in a buffer (100 mM Tris-HCl, pH 7.5, 5 M NaCl, 0.5 m EDTA, 10% SDS, 1% NP40, IGEPAL) containing protease and phosphatase inhibitors and centrifuged at 15,000× *g* for 20 min at 4 °C. The supernatant was collected, and protein concentration was determined using a protein assay kit (Sigma-Aldrich). Samples containing 40 μg of total proteins were solubilized and electrophoresed on a 12% sodium dodecyl sulphate-(SDS) polyacrylamide gel. Following electrophoresis, proteins were transferred to a nitrocellulose membrane (Bio-Rad Laboratories, Milan, Italy). The membrane was immersed in a blocking solution (3% non-fat dried milk in 20 mM Tris and 137 mM NaCl at pH = 7.6 containing 0.05% Tween-20) for 2 h on a plate shaker. Subsequently, the membrane was incubated overnight at 4 °C on the plate shaker with mouse anti-βIII tubulin (1:1000; Abcam) primary antibody. Blot was probed with horseradish peroxidase-labeled anti-mouse secondary antibody, and the bands were visualized with enhanced chemo luminescence reagents (Bio-Rad Laboratories). To check for equal loading of the gel, membranes were probed with rabbit anti-GAPDH (glyceraldehyde 3-phosphate dehydrogenase, 1:2000, Sigma-Aldrich).

Image analysis was carried out by ChemiDoc System (Bio-Rad Laboratories).

The intensity of the blotting was measured using the software Image J and is given as the mean ± S.E.M. of the optical density for each experimental group, calculated in three or four independent experiments.

### 2.12. Transmission Electron Microscopy

PC12 cells were centrifuged at 1000× *g* for 5 min, and the supernatant was removed. Cell pellet was fixed in 2.0% paraformaldehyde and 0.1% glutaraldehyde in 0.1 M PBS, pH 7.4 for 90 min at 4 °C. U87MG cells, after removing the culture medium, were fixed with the same fixing solution (for 90 min at 4 °C), gently scraped from the plate, and centrifuged at 10,000× *g* rpm for 10 min to obtain the cell pellet.

This fixing solution contained a concentration of aldehyde, which minimally covers antigen epitopes while fairly preserving tissue architecture. After washing, specimens of both cell lines were post-fixed in 1% OsO_4_ for 1 h at 4 °C; they were dehydrated in ethanol and finally embedded in epoxy resin.

Plain TEM was implemented by a post-embedding immunocytochemistry procedure for antibodies against LC3 (Abcam, Cambridge, UK) and LAMP1 (Gene Tex, Irvine, CA, USA) used as markers of autophagy vacuoles (autophagosomes and autophagolysosomes, respectively), according to the manuscript “Guidelines for the Use and Interpretation of Assays for Monitoring Autophagy (4th Edition)” [87].

At the end of plain TEM or immunocytochemistry procedure, ultrathin sections were stained with uranyl acetate and lead citrate, and they were finally examined using a JEOL JEM-100SX transmission electron microscope (JEOL, Tokyo, Japan).

### 2.13. Post-Embedding Immunocytochemistry

Fixing and post-fixing solutions as well as epoxy resin were validated in previous studies for immuno-gold-based ultrastructural morphometry [47,68,88].

Post-embedding procedure was carried out on ultrathin sections collected on nickel grids, which were incubated on droplets of aqueous sodium metaperiodate (NaIO_4_) for 30 min at room temperature to remove OsO_4_. NaIO_4_ is an oxidizing agent allowing close contact between antibodies and antigens by removing OsO_4_ [70]. This step allows a better visualization of immuno-gold particles specifically located within a sharp context of cell integrity, in order to count molecules within specific cell compartments [46,47,68]. After washing in PBS, the grids were incubated in a blocking solution containing 10% goat serum and 0.2% saponin for 20 min at room temperature. Grids were then incubated with primary antibody solutions containing rabbit anti-LC3 (Abcam, diluted 1:50) or mouse anti- LAMP1 (1:30) antibodies with 0.2% saponin and 1% goat serum in a humidified chamber overnight, at 4 °C. After washing in PBS, grids were incubated with the secondary antibodies conjugated with gold particles (20 or 10 nm mean diameter, BB International), diluted 1:20 in PBS containing 0.2% saponin and 1% goat serum for 1 h, at room temperature. Negative control sections of immunocytochemistry reactions were incubated with the secondary antibody only.

### 2.14. Ultrastructural Morphometry

For ultrastructural morphometry, grids containing non-serial ultrathin sections (70–90 nm thick) were counted at TEM 8000 ×.

In these sections, the number of both total and altered mitochondria and the number of LC3 and/or LAMP1 positive vacuoles were counted in a total of 50 cells/group.

Mitochondria were easily identified at TEM for their shape and structure. They possess a typical double-membrane, which limits an inter-membrane space and an area internal to the inner membrane, containing a homogeneous matrix regularly interrupted by intermingled crests (cristae). Although the basic ultrastructural morphology of mitochondria is well standardized, variations can occur during functional activities in physiological and pathological states. Ultrastructural criteria used here to identify altered mitochondria were defined in detail in Ferese et al., (2020) [84].

To count LC3 and/or LAMP1 positive vacuoles, cell vacuoles possessing single, double, or multiple membranes and the same electron density of the surrounding cytoplasm (or partly containing some electron dense structure) were considered according with Klionsky et al., (2021) [87].

### 2.15. Statistical Analysis

For cell viability experiments, WST-1 activity is given as the mean percentage ± S.E.M. percentage of optical density (assuming control as 100% WST-1) calculated in three independent experiments. Values obtained in TB experiments are given as the percentage of TB-positive cells ± S.E.M. percentage counted in three independent experiments. For H&E experiments, number of stained cells detectable after each specific treatment is given as the mean percentage ± S.E.M. percentage (assuming control as 100%) of cell counts carried out at 20× magnification in three independent experiments. The number of FJB-positive cells was counted at 20× magnification in three independent experiments. Values are given as the mean number ± S.E.M. of FJB-positive cells.

FJB, Mito Tracker Red, and Mito Tracker Green staining, intensity of the fluorescence was measured in 50 cells/group obtained from three independent experiments and are given as the mean percentage ± SEM percentage (assuming control as 100%). 

For Western blot assay, optical density values are given as the mean ± S.E.M. of optical density measured in four independent experiments.

Data collected for ultrastructural morphometry were counted in 50 cells per group and are given as an absolute number per cell concerning: (i) autophagy-like vacuoles, (ii) LC3 and/or LAMP1 positive vacuoles, (iii) LC3 or LAMP1 immuno-gold particles. Furthermore, we used ratios to express (i) the number of LC3 or LAMP1 immuno-gold particles within vacuoles out of the number of cytoplasmic LC3 or LAMP1 immuno-gold particles. Values are given as the mean ± S.E.M. per cell or the mean percentage ± S.E.M. percentage.

Total and altered mitochondria were counted from 50 cells per group. The number of mitochondria was given as the mean ± S.E.M. per cell. The amount of altered mitochondria is given as the mean percentage of altered mitochondria ± S.E.M percentage.

All data collected for each group were compared by using one-way analysis of variance, ANOVA, followed by Scheffè’s post hoc analysis. Differences between groups were considered significant when the null hypothesis (H_0_) was *p* ≤ 0.05.

## 3. Results

### 3.1. Dose- and Time-Dependent Effects of LF on PC12 Cell Viability

**Figure 1:**
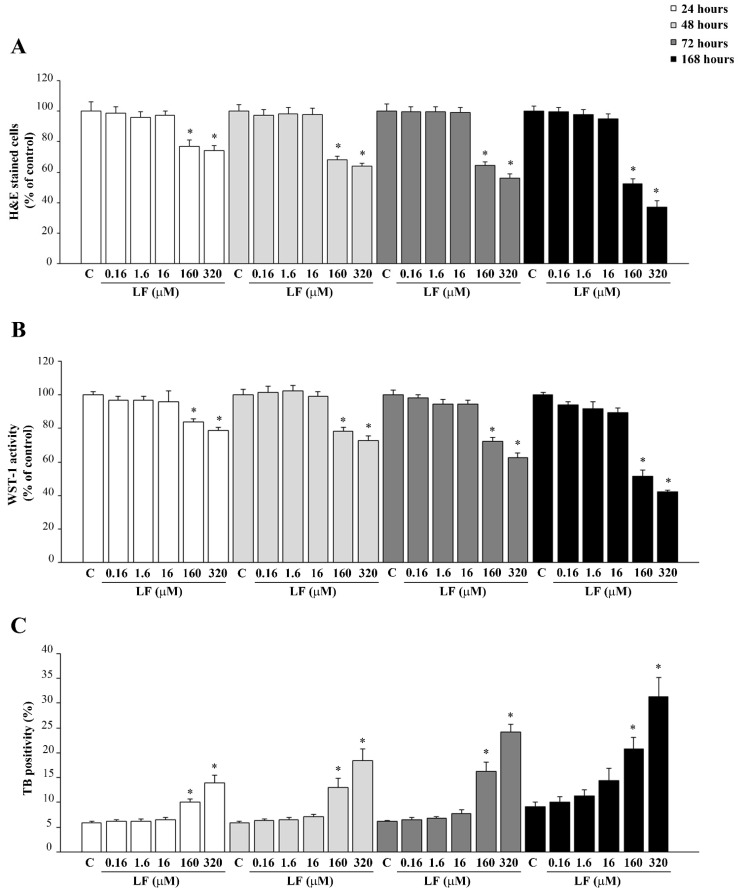
Dose–response and time-course of LF on cell viability. Time and dose–response effects of LF on cell viability; (**A**) H&E, (**B**) WST-1 assay, and (**C**) TB staining. C (control) refers to untreated P12 cells. * *p* < 0.05 compared with control.

In the first group of experiments, the effects of LF on PC12 cell viability were evaluated. Various doses of LF (ranging from 0.16 μM to 320 μM) were administered at various time intervals (ranging from 24 h up to 168 h). As shown in Figure 1, low doses of LF, up to 16 μM, do not affect PC12 cell viability at any time of exposure, while high doses of LF (160 μM and 320 μM) time-dependently reduce cell survival. This is assessed through different procedures, consisting in H&E staining (Figure 1A), WST-1 assay (Figure 1B) and TB staining (Figure 1C). Representative images of H&E-stained PC12 cells related to not toxic (16 μM) or frankly toxic LF doses (320 μM) are reported in Supplementary Figures S1 and S2, respectively.

**Figure 2:**
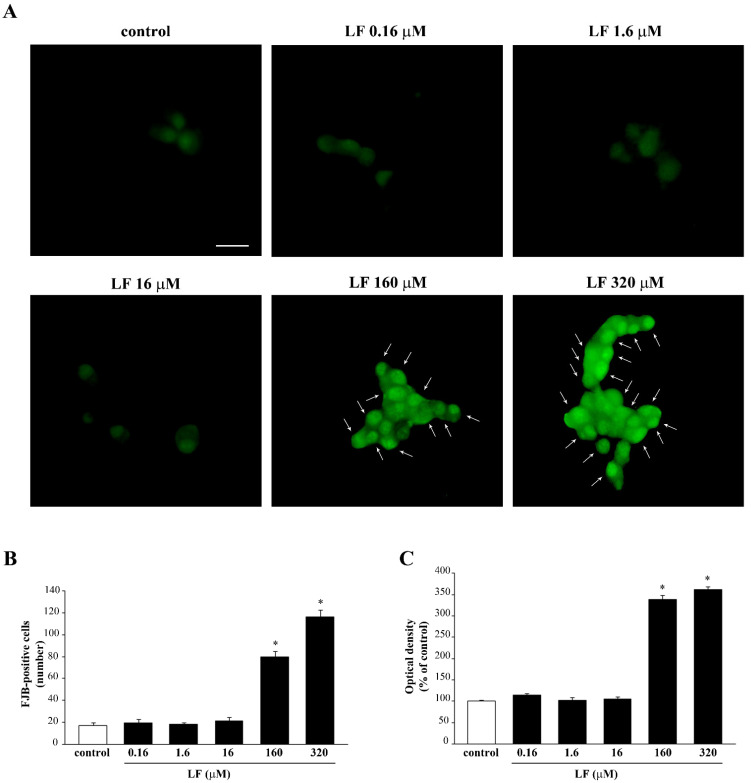
Dose–response of LF on FJB staining. (**A**) Representative pictures of FJB-stained PC12 cells after treatment with various doses (from 0.16 μM up to 320 μM) of LF for 72 h. The number and intensity of FJB fluorescent cells is reported in graphs (**B**,**C**), respectively. Arrows indicate FJB intensely positive cells. * *p* < 0.05 compared with control. Scale bar = 17 μM.

The consistency of results obtained with different methods allows selecting which LF dose was the most appropriate and the best time of exposure. This extends to the fluorescent dye FJB (Figure 2A–C). In fact, consistently with previous results, only the high doses of LF (160 μM and 320 μM) increase FJB fluorescence, while low doses of LF do not differ from controls (Figure 2A–C). In detail, the LF doses of 1.6 μM and 16 μM and the time interval of 72 h were selected, respectively, to investigate the effects of LF on METH-induced toxicity. Similar dose–response of LF on FJB staining were obtained in the negative control U87MG cells (Supplementary Figure S3). 

### 3.2. LF Induces Elongation of PC12 Cells

**Figure 3:**
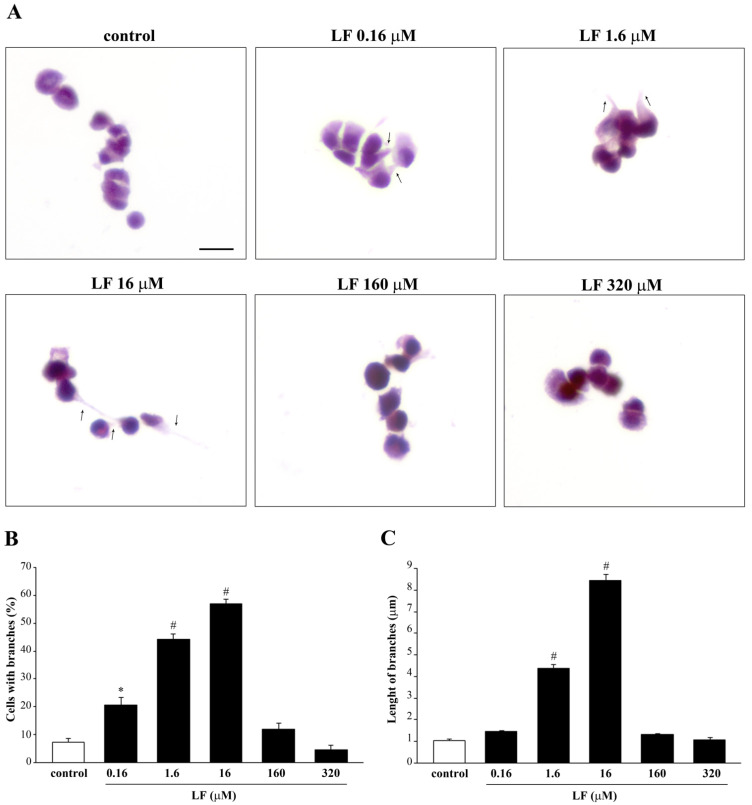
Morphological changes induced by LF in PC12 cells. (**A**) Representative pictures of H&E-stained PC12 cells after treatment with LF ranging from 0.16 μM up to 320 μM for 72 h. Cell branches (arrows) are induced by LF. The histograms report (**B**) the mean number of cells with branches and (**C**) the mean branch length for each dose of LF. * *p* < 0.05 compared with control; # *p* < 0.05 compared with other groups. Scale bar = 15 μM.

Analysis of H&E-stained PC12 cells at light microscopy indicates that LF administration induces cell branching (Figure 3A). These morphological changes are evident following low LF doses, up to 16 μM, but are no longer present in PC12 cells treated with high LF doses (160 μM and 320 μM).

Remarkably, both dose response curve for the number of cells with branches and the length of branches are reverse U shaped. They both increase following increasing doses of LF, reaching a peak at the dose of 16 μM LF (Figure 3B,C), while at high LF doses (160 μM and 320 μM), both number and length of cell branches decrease (Figure 3). Interestingly, LF induces cell branches also in U87MG cells (Supplementary Figure S4). 

### 3.3. LF Reduces METH-Induced Toxicity in PC12 Cells

**Figure 4:**
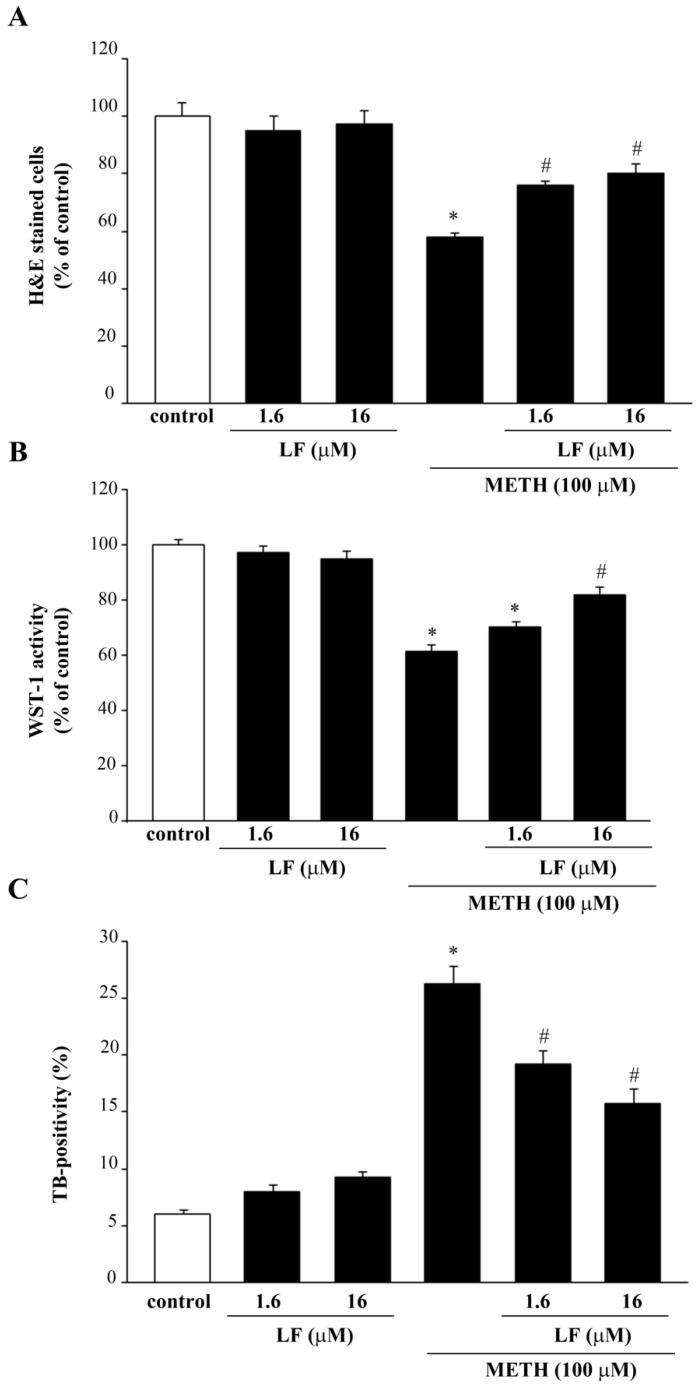
LF reduces METH-induced toxicity in PC12 cells. Assessment of PC12 cells viability through (**A**) H&E staining, (**B**) WST-1 assay, and (**C**) TB staining following administration of METH, alone or in combination with LF at 1.6 μM and 16 μM for 72 h. * *p* < 0.05 compared with control; # *p* < 0.05 compared with control and METH.

**Figure 5:**
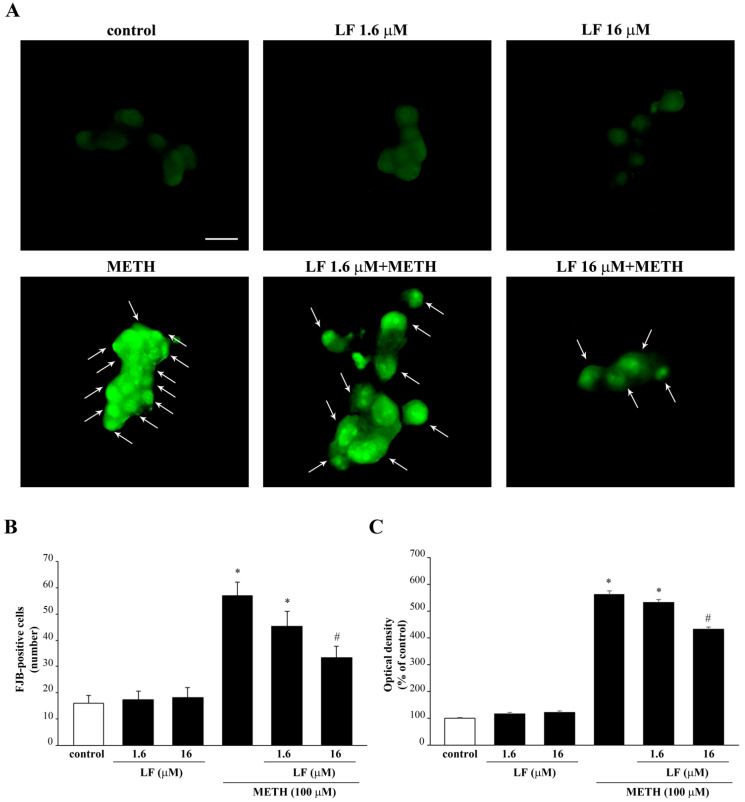
LF decreases METH-induced FJB-responsiveness in PC12 cells. (**A**) Representative pictures of FJB-stained PC12 cells after combined LF and METH treatment. The graphs (**B**,**C**) report the number and the intensity of FJB fluorescent cells, respectively. Arrows indicate FJB intensely positive cells. * *p* < 0.05 compared with control; # *p* < 0.05 compared with control and METH. Scale bar = 15 μM.

METH administration at the dose of 100 μM produces moderate cell loss, as previously published [48]. This is assessed by H&E, WST-1, and TB (Figure 4), and is further confirmed by FJB fluorescence (Figure 5). Since the effects of LF are supposed to occur ubiquitously in each cell phenotype, it is crucial to assess whether these ubiquitous effects turn to be neuroprotective when exerted in METH susceptible cell phenotype. The reduction in cell viability produced by METH is partially preserved by pre-treatment with LF (Figure 4). Consistently, cell death measured by TB shows that METH increases the TB-stained cells, which is attenuated by LF pre-treatment (Figure 4). 

Again, administration of LF reduces METH-induced increase in FJB-fluorescence, thus indicating a protective effect of LF on METH toxicity (Figure 5). This is evident in the intense FJB-responsiveness of METH-treated cells, as indicated by arrows (Figure 5). This effect is attenuated dose-dependently when PC12 cells are pre-treated with LF, as indicated by a reduction in intensely FJB-positive cells (arrows, Figure 5). Protection is remarkable for the doses of 1.6 μM and 16 μM LF, which never fully prevent cell loss induced by METH (Figure 4 and Figure 5). 

As expected, when METH was administered to U87MG cells, which do not contain catecholamine including DA, no METH toxicity was observed (Supplementary Figures S5 and S6). 

### 3.4. Combined Exposure to LF and METH Enhances Phenotypic Changes in PC12 Cells

**Figure 6:**
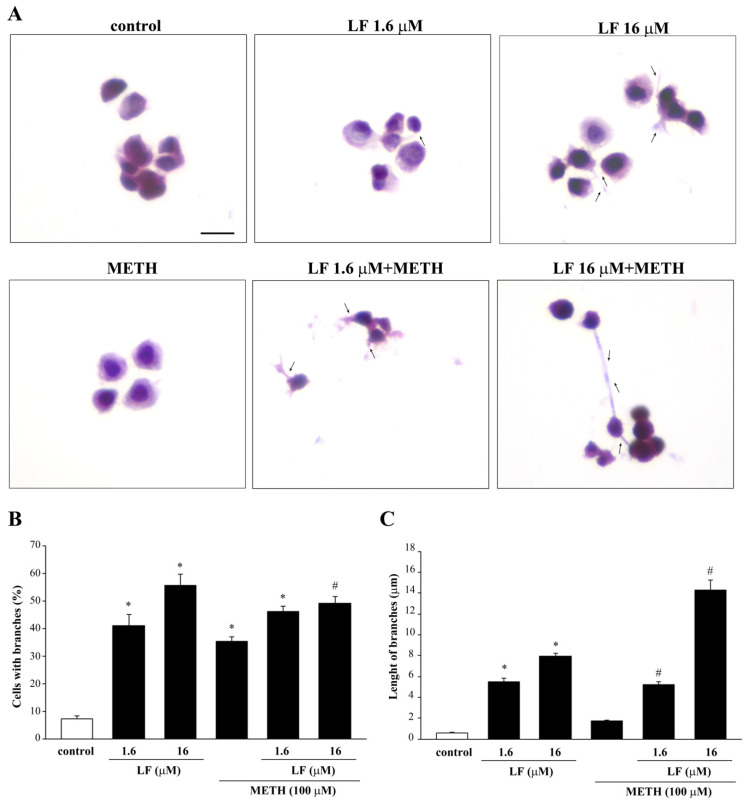
METH enhances the growth of cell elongations induced by LF. (**A**) Representative pictures of H&E-stained branching out of PC12 cells after combined LF and METH treatment. Arrows indicate cell elongations. The histograms report (**B**) the mean number of cells with branches and (**C**) the mean length of branches observed in each experimental group. * *p <* 0.05 compared with control; # *p <* 0.05 compared with METH. Scale bar = 12 μM.

H&E staining was used to analyze the phenotypic effects of a combined LF and METH treatment (Figure 6A). Differing from LF alone, administration of METH alone induces the growth of cell branches without affecting branch length. When LF and METH are co-administered, the length of cell branching increases compared with single treatments (Figure 6), while the mean number of cells with branches is similar in the combined treatment and LF alone, both surpassing the number of branched cells induced by METH alone. In detail, in baseline conditions (control), only 7.3 ± 0.9% of cells possess short branches (Figure 6B), which measure 0.6 ± 0.1 μM (Figure 6C). Treatment with LF 16 μM increases the percentage of cells with branches to 55.7 ± 4.2% (Figure 6B) along with their length, which reaches 8.0 ± 0.3 μM (Figure 6C). Combined treatment with METH and LF 16 μM leads the length of cell branches up to 14.3 ± 1.0 μM (Figure 6C), while the percentage of cells with branches remains steady (49.3 ± 2.3%, Figure 6B).The lower dose of LF administered either alone or in combination with METH, produces an increase in the percentage of cells with branches (41 ± 4.2% or 46.3 ± 1.9%, respectively), and their length (5.5 ± 0.3 μM or 5.2 ± 0.3 μM). As expected, METH does not produce any phenotype change in U87MG cells, and it does not influence the effect on cell branches produced by LF 16 μM (Supplementary Figure S7). 

**Figure 7:**
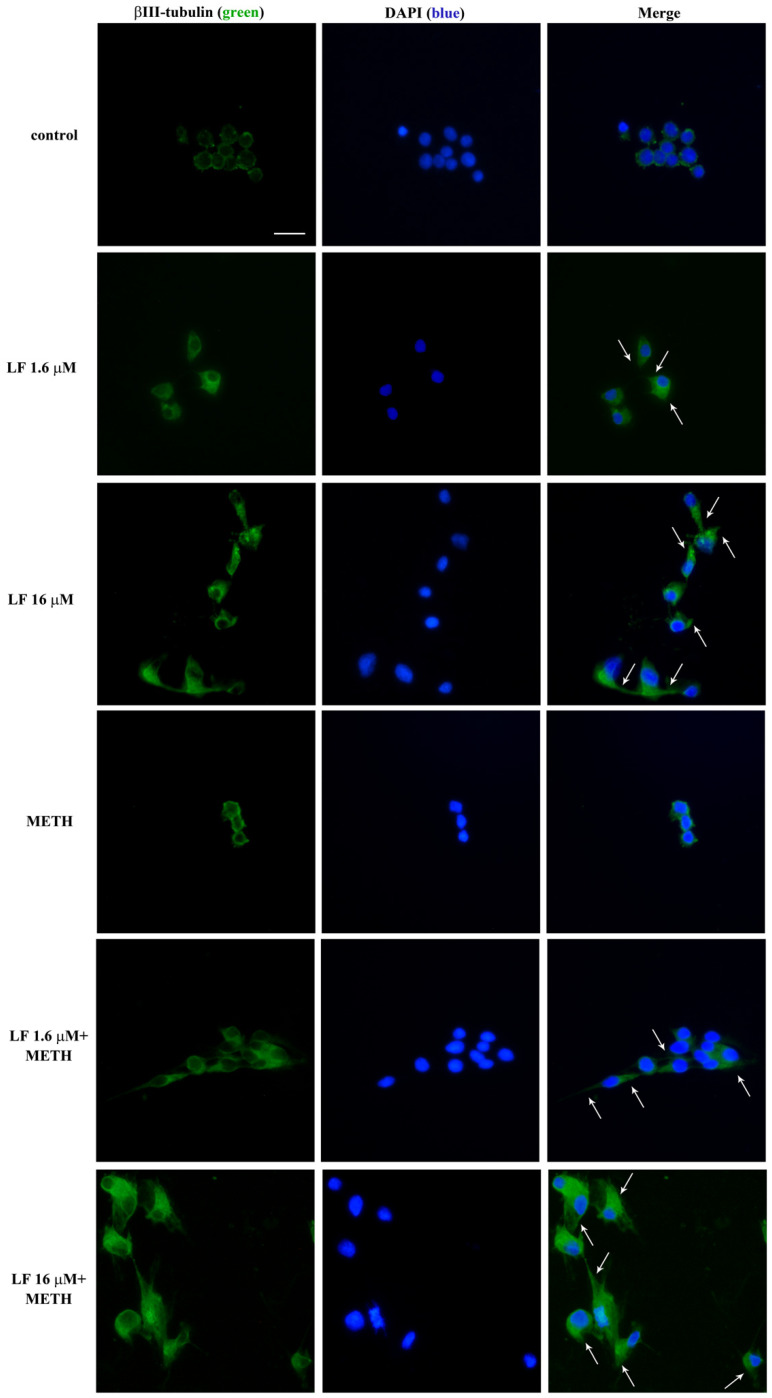
Representative βIII-tubulin immuno-fluorescence. Representative pictures of βIII-tubulin immuno-fluorescence after single and combined LF (1.6 μM and 16 μM) and METH administration. Arrows indicate βIII-tubulin immuno-fluorescent cell branches. Scale bar = 16 μM.

**Figure 8:**
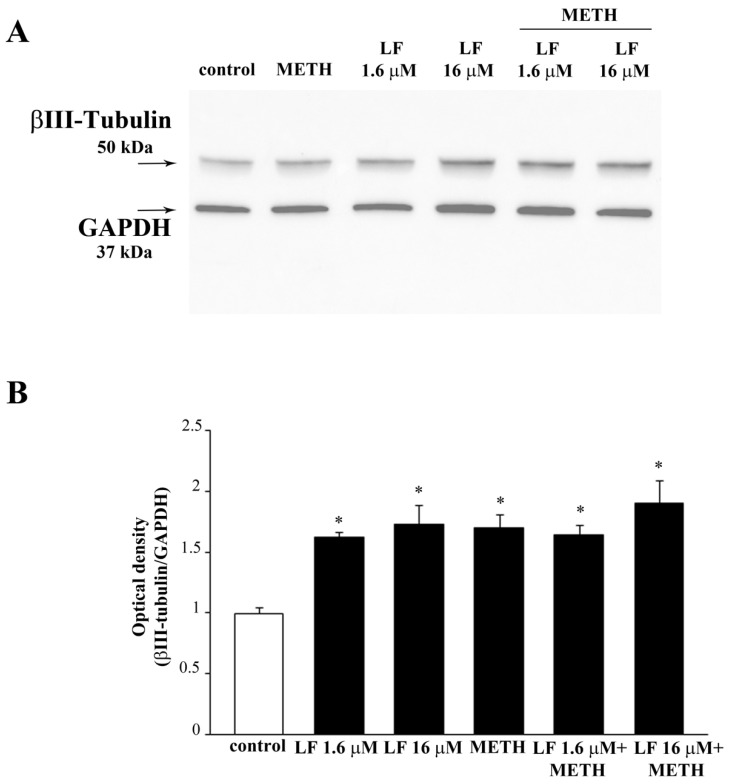
βIII-Tubulin Western blotting. (**A**) Representative Western blotting for the neuro-filament marker βIII-tubulin in control cells and in cells treated with LF and METH, alone or in combination. (**B**) The graph reports the ratio between the optical density of βIII-tubulin and the housekeeping protein GAPDH. GAPDH was selected as the housekeeping protein to avoid the bias to measure another neuro-filament maker (β-actin) as a housekeeping protein. Still, a quite similar biochemical modulation between GAPDH and neuro-filament expression may explain why the results of Western blotting are flattened between various groups compared with immuno-fluorescence. * *p* < 0.05 compared with control.

In a further set of experiments, the expression of the neuro-filament antigen βIII-tubulin is analyzed by immuno-fluorescence and Western blot (Figure 7 and Figure 8, respectively). Consistently with the effects on cell branching (Figure 6), both doses of LF, either alone or in combination with METH, increase βIII-tubulin (Figure 7 and Figure 8). In detail, LF per se induces the growth of βIII-tubulin immuno-fluorescent cell branches, as indicated by arrows in Figure 7. Remarkably, this effect is neither suppressed nor attenuated by METH (Figure 7). These findings are in line both with increased percentage of cells with branches and the length of cell branches, which occurs following LF, at both doses, alone or combined with METH (Figure 6). The same results are obtained by Western blotting βIII-tubulin (Figure 8A,B). Expression of βIII-tubulin is analyzed also in U87MG cells, both by immunofluorescence (Supplementary Figure S8) and Western blot (Supplementary Figure S9).

### 3.5. LF Increases Healthy Mitochondria while Counteracting METH-Induced Mithocondrial Alterations

**Figure 9:**
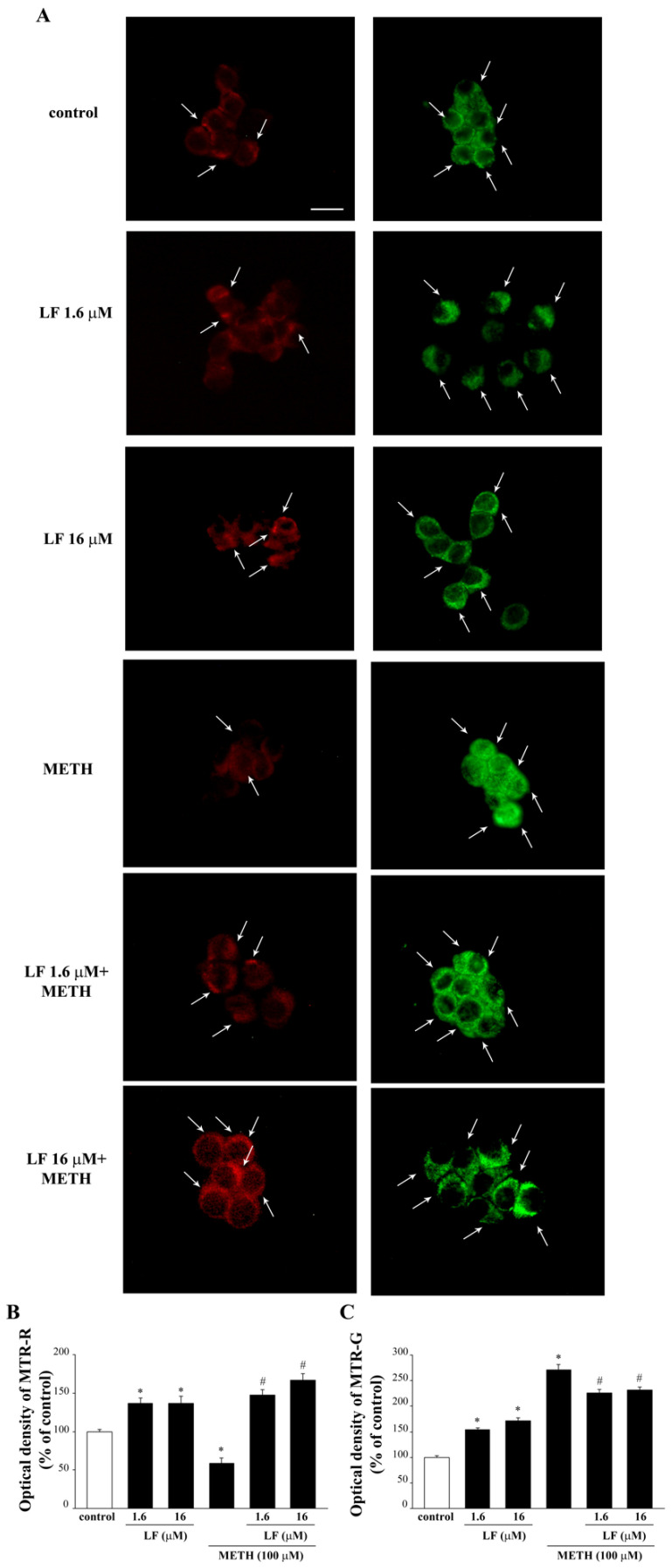
Mito Tracker-fluorescence after combined METH and LF administration. (**A**) Representative pictures of Mito Tracker Red and Mito Tracker Green fluorescent cells, which label healthy and total mitochondria, respectively. Arrows indicate intensely MTR-R or MTR-G-stained cytosolic spots. Optical density of Mito Tracker Red (**B**) and Mito Tracker Green (**C**). MTR-R = Mito Tracker Red; MTR-G = Mito Tracker Green. * *p <* 0.05 compared with control; # *p* *<* 0.05 compared with METH. Scale bar = 13 μM.

As hypothesized, LF does produce beneficial effects on mitochondria when administered alone. In fact, as evident from representative Figure 9A and graph of Figure 9B, LF significantly increases the amount of MTR-R immuno-fluorescence at both doses, compared with controls. Such an increase in MTR-R indicates the number of healthy mitochondria and it corresponds to the increase in MTR-G showing the total mitochondria. This implies that the increase in total mitochondria produced by both doses of LF corresponds to healthy mitochondria. In sharp contrast, when METH is administered alone, a decrease in MTR-R is observed and measured (Figure 9A,B, respectively), while an increase in MTR-G occurs (Figure 9A,C, respectively). This indicates that METH increases the total number of mitochondria while decreasing healthy mitochondria compared with controls. This is indicated by the decrease in intensely MTR-R-stained cytosolic spots (arrows, Figure 9A) and the concomitant increase in intensely MTR-G-stained cytosolic spots (arrows, Figure 9A) observed in METH-treated cells. Thus, METH slightly decreases healthy mitochondria and markedly increases total mitochondria. This suggests that the increase in the number of damaged mitochondria is severe. When LF is administered before METH, the increase in MTR-R is fully restored (Figure 9A,B), while the increase in total mitochondria is less pronounced compared with METH alone. This indicates that the increase in healthy mitochondria parallels what measured following LF alone (Figure 9B). This suggests that the increase in altered mitochondria is abolished when METH is administered to LF pre-treated cells.

**Figure 10:**
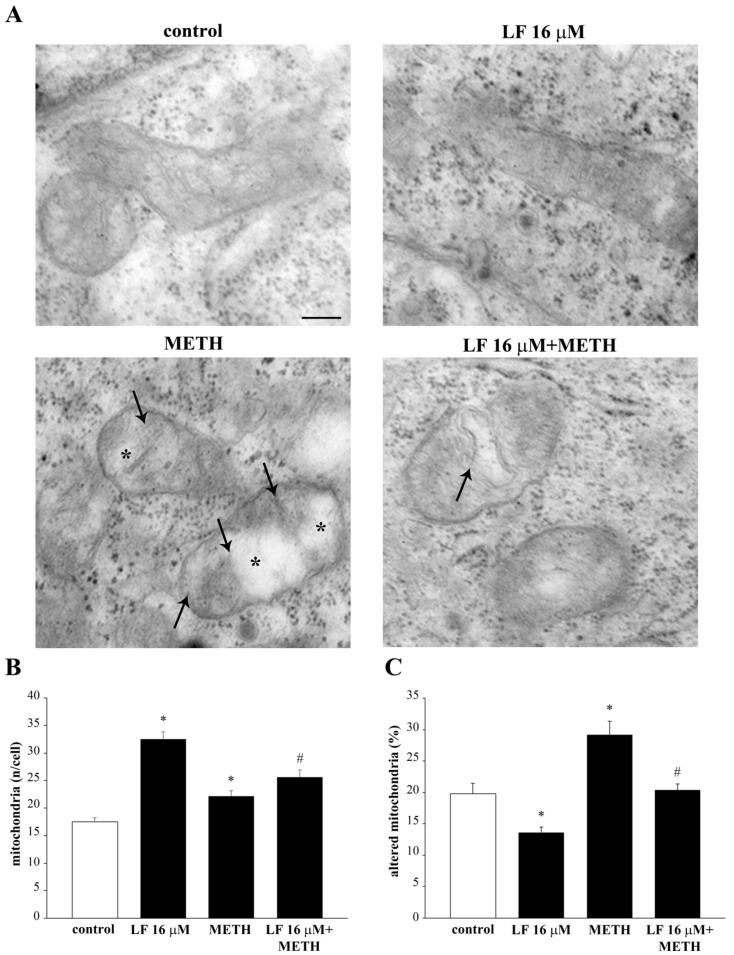
LF prevents METH-induced alteration of mitochondrial structure and number. (**A**) Representative micrographs showing mitochondria from a control cell and from cells treated with either LF, or METH, or combined LF + METH. Note the ultrastructure of well-preserved mitochondria from controls and LF-treated cells. After, METH mitochondria show crest fragmentation (arrows) and matrix dilution (*). These effects are attenuated by previous LF administration, which mitigates mitochondrial crest derangement (arrow). The histograms report (**B**) the number of mitochondria per cell and (**C**) the percentage of altered mitochondria following various treatments. * p < 0.05 compared with control; # *p* < 0.05 compared with METH. Scale bar = 90 nm.

Ultrastructural analysis at TEM confirms these findings (Figure 10). In fact, LF at the dose of 16 μM leads to a two-fold increase in the total number of mitochondria (Figure 10A,B). This effect is sustained by healthy mitochondria, as represented in Figure 10A, showing a small, well-shaped, electron-dense organelle, where crests are well defined within an electron-dense matrix. In fact, the number of altered mitochondria, which were counted after such a dose of LF, were robustly decreased (Figure 10C). In cells administered METH alone, the mitochondrial status at electron microscopy was dramatically worsened, as shown in representative Figure 10A, where a large, stagnant, non-homogeneous mitochondrion is evident, owing to an internal structure with minimal electron density and a lack of defined crest architecture. This is counted in graphs of Figure 10B,C, reporting an increase in total mitochondria with a dramatic increase in the number of altered organelles. The pre-administration with LF partially occludes the deleterious action induced by METH on mitochondrial status and number of altered mitochondria (Figure 10A–C). This indicates how specific is LF in counteracting METH-induced mitochondrial alterations. 

### 3.6. LF Promotes LC3 Compartmentalization and Reverses METH-Induced LC3 Dissipation

**Figure 11:**
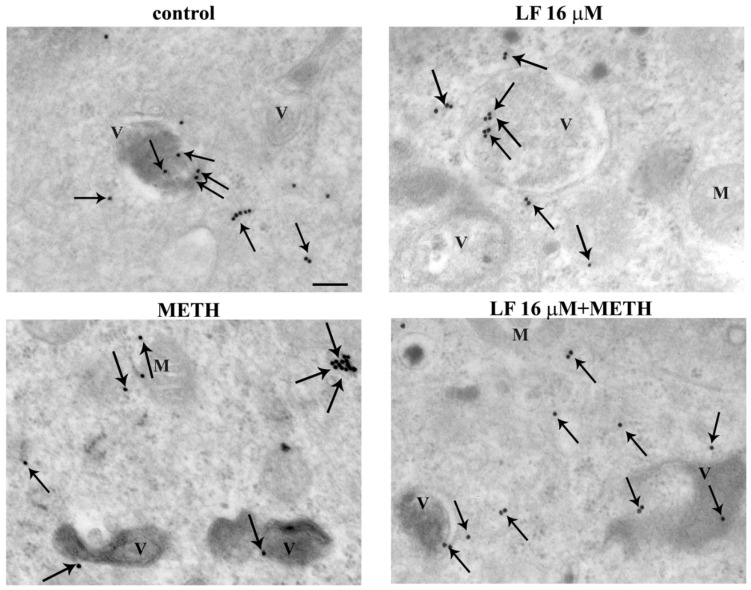
LF increases LC3 compartmentalization within vacuoles. Representative TEM pictures of LC3 positive vacuoles from a control cell and cells treated with either LF 16 μM or METH or LF + METH. Arrows point to LC3 immuno-gold particles. V = vacuoles; M = mitochondria. Scale bar = 125 nm.

**Figure 12:**
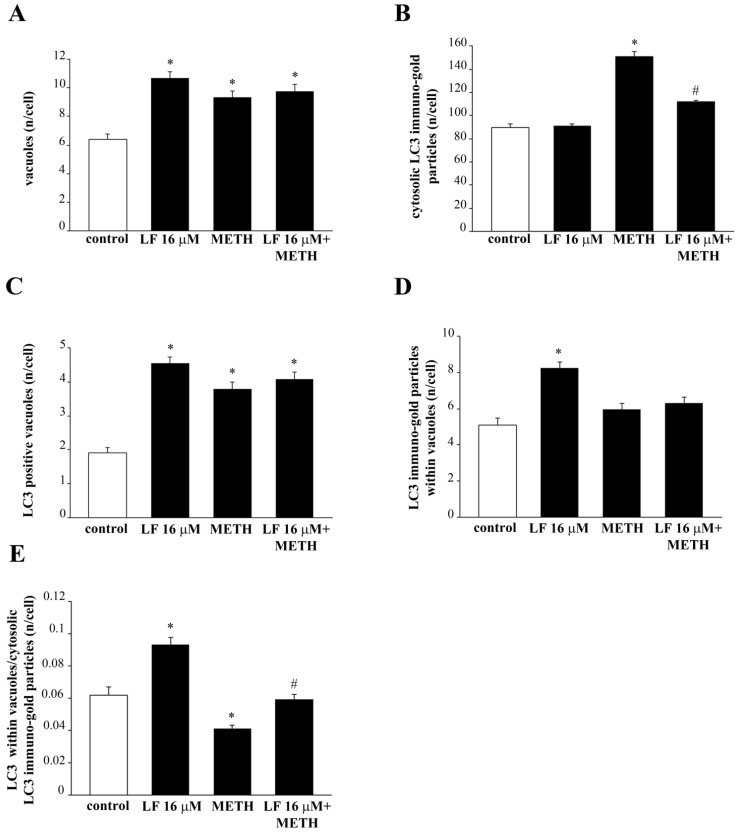
LF increases LC3 compartmentalization within vacuoles. Quantitative ultrastructural morphometry for LC3 is reported in the graphs (**A**–**E**). * *p* < 0.05 compared with control; # *p* < 0.05 compared with METH.

METH toxicity and mitochondrial alterations are both tightened to an altered autophagy status. In fact, METH toxicity is characterized a defective autophagy [46,68]. At first, the autophagy was grossly approached by measuring the expression of the specific autophagy proteins LC3, as shown in the representative picture of Figure 11. The measurement of vacuoles (Figure 12A), whole cell LC3 particles (Figure 12B), LC3 positive vacuoles (Figure 12C), LC3 particles within vacuoles (Figure 12D), and the ratio between compartmentalized (vacuolar) vs. cytosolic LC3 particles (Figure 12E), were carried out by ultrastructural stoichiometry. As shown in Figure 12A, LF alone produces a marked increase in the number of vacuoles. Such an amount, which is almost two-fold of controls, is reproduced by METH alone and LF + METH. When the amount of cytosolic LC3 was measured, LF did not modify the control value, whereas METH increased the number of LC3 particle in the whole cell (Figure 12B). The pre-treatment with LF greatly suppresses the increase in cytosolic LC3 produced by METH (Figure 12B). Remarkably, when the amount of LC3 positive vacuoles and LC3 immuno-gold particles were measured, LF produced a marked increase, which exceeded that measured in controls (Figure 12C,D). When the ratio of vacuolar vs. cytosolic LC3 was calculated, LF alone produced a marked LC3 compartmentalization within vacuoles compared with controls. Contrarily, METH dissipates LC3 from vacuoles to cytosol. The concomitant administration of LF and METH brought back LC3 compartmentalization to control values (Figure 12E). This indicates that the deleterious dissipation of LC3 from vacuoles, which is key in the autophagy derangement induced by METH [49], was occluded by pre-administering LF, which, by itself, sustains an over-compartmentalization of LC3, which suggests a bigger reservoir of efficient autophagy vacuoles in the cell. Similarly, LF increases the number of vacuoles, the amount of LC3 positive vacuoles and LC3 immuno-gold particles measured within the vacuoles, as well as the ratio of vacuolar vs. cytosolic LC3 in U87MG cells, thus demonstrating that also in this cell line LF alone produces a compartmentalization of LC3 particles within vacuoles compared with controls (Supplementary Figure S10).

Accordingly, in this cell line, LF continues to exert its ubiquitous effects on autophagy proteins. However, this is not related to neuroprotection; in fact, this cell line is not susceptible to METH toxicity. This demonstrates that, despite being ubiquitous, the effects of LF are neuroprotectant in specific cell phenotypes. 

### 3.7. LF Promotes the Merging of LC3 with Cathepsin D

**Figure 13:**
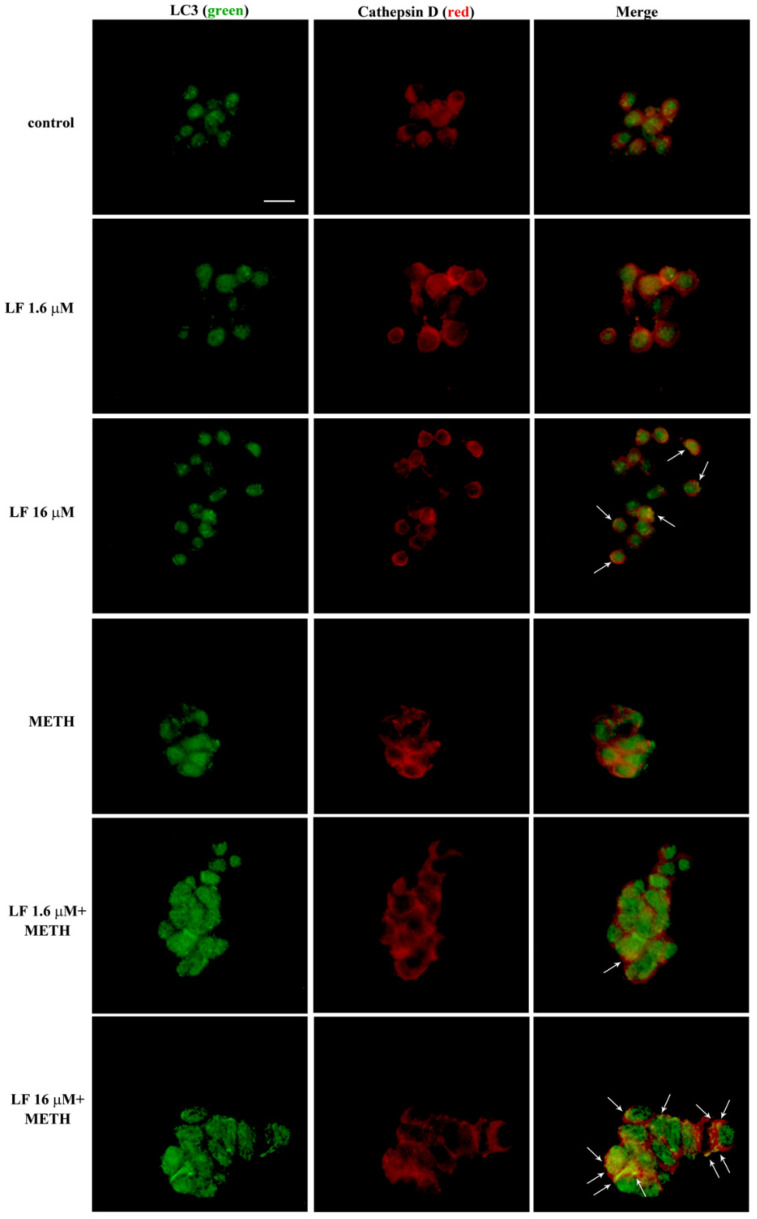
LF increases LC3 and Cathepsin D co-localization. Representative pictures showing double immuno-fluorescence for LC3 (green) and Cathepsin D (red), and their merge in combined LF and METH treated cells. Arrows indicate cells with intensely merged fluorescence resulting in yellow puncta. Scale bar = 15 μM.

In order to test the real efficiency of these autophagy vacuoles, the merging between LC3 and Cathepsin D is shown in Figure 13, which indicates that LF alone increases the merging of LC3 with Cathepsin D, while this is occluded by METH compared with controls. Pre-treatment of LF restores the merging of LC3 with Cathepsin D. This is mostly evident for the 16 μM dose of LF (Figure 13).

### 3.8. LF Promotes the Merging of LC3 with LAMP1 and Promotes the Fusion of Autophagosomes with Lysosomes

**Figure 14:**
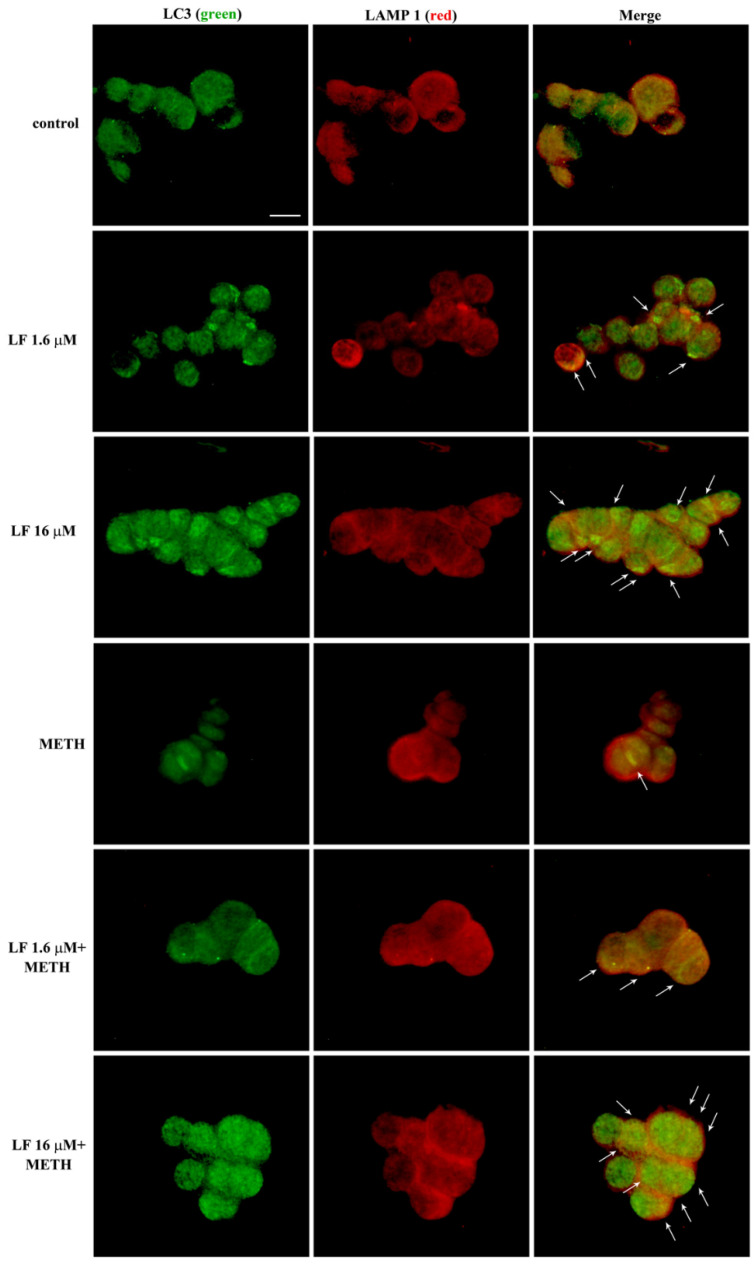
LF increases LC3 and LAMP1 co-localization. Representative double immuno-fluorescence for LC3 (green) and LAMP1 (red), and their merge in combined LF and METH treated cells. Arrows indicate cells with intensely merged fluorescence, resulting in yellow puncta. Scale bar = 12 μM.

**Figure 15:**
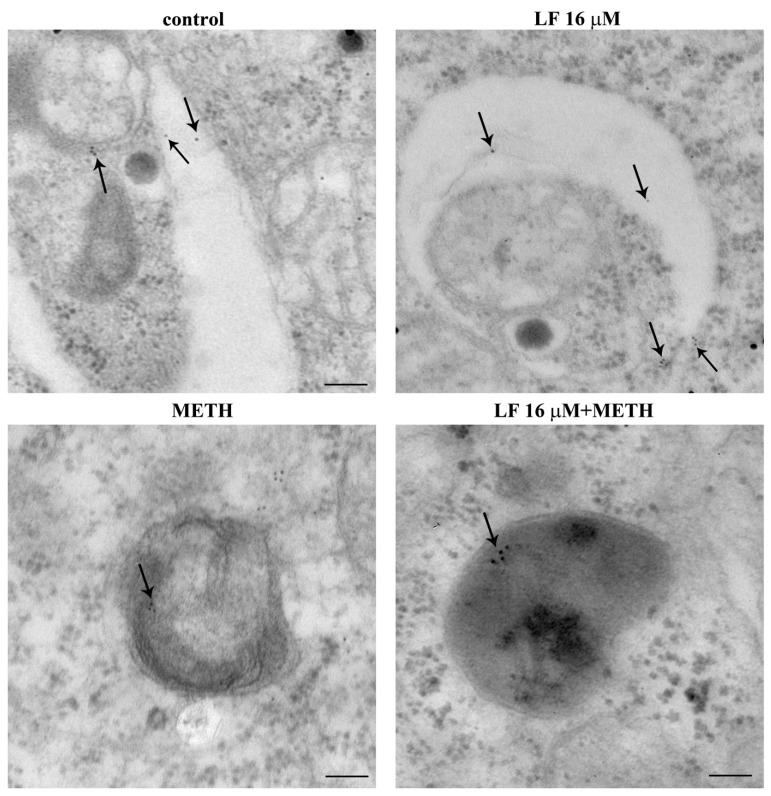
Lactoferrin increases LAMP1 compartmentalization within vacuoles. Representative TEM pictures of LAMP1 positive vacuoles from a control cell, and cells treated with either LF 16 μM or METH or LF + METH. Arrows point to LAMP1 immuno-gold particles within vacuoles. Scale bar = 100 nm (control and LF 16 μM); 200 nm (METH); 65 nm (LF 16 μM + METH).

To further document the authentic significance of LF in committing autophagosomes, Figure 14 shows the merging between LC3 and another lysosome-related antigen, LAMP1 by immuno-fluorescence. This is further shown at electron microscopy in Figure 15. These data recapitulate what shown in representative Figure 13. In detail, Figure 14 shows that LF, when co-administered with METH, restores the merging of LC3 with LAMP1 observed in control.

**Figure 16:**
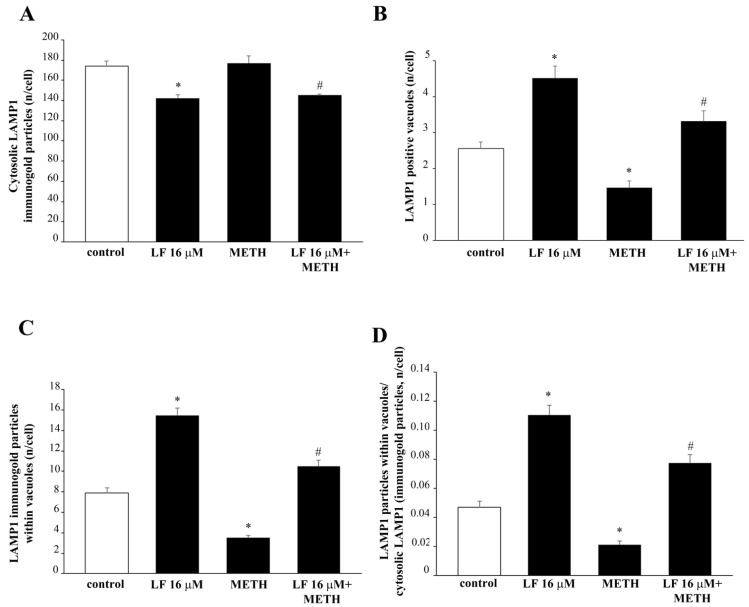
Lactoferrin increases LAMP1 compartmentalization within vacuoles. Graphs (**A**–**D**) report the ultrastructural morphometry for LAMP1. (**A**) = * *p* <0.05 compared with control; # *p* < 0.05 compared with control and METH; (**B**) = * *p* < 0.05 compared with control; # *p* < 0.05 compared with METH (**C**) = * *p* < 0.05 compared with control; # *p* < 0.05 compared with METH; (**D**) = *p* < 0.05 compared with control; # *p* < 0.05 compared with METH.

**Figure 17:**
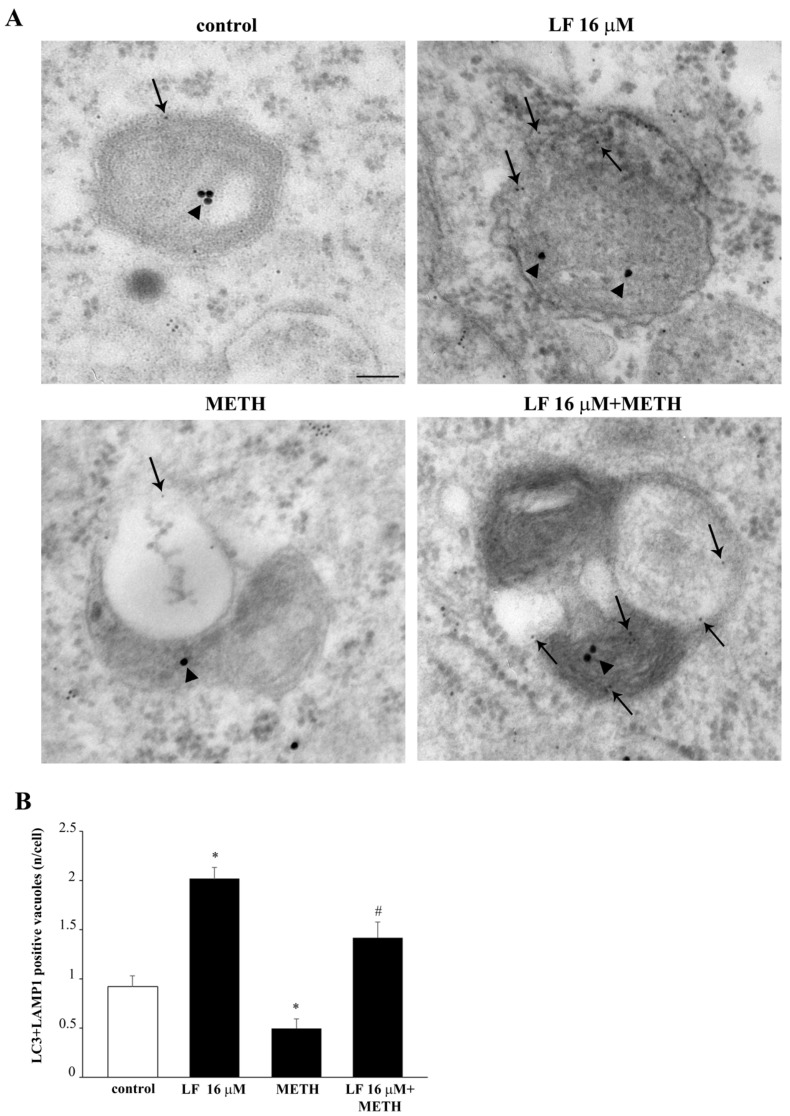
LF increases LC3 and LAMP1 co-localization within vacuoles. (**A**) Representative TEM pictures of LC3 and LAMP1 positive vacuoles from a control cell, and cells treated with either LF 16 μM or METH or LF + METH. Arrowheads and arrows point to LC3 (20 nm) and LAMP1 (10 nm) immuno-gold particles within vacuoles, respectively. The graph (**B**) reports the number of vacuoles double-stained with LC3 and LAMP1 immuno-gold particles. * *p* < 0.05 compared with control; # *p* < 0.05 compared with METH. Scale bar = 100 nm.

When ultrastructural stoichiometry is carried out within specific autophagosome and lysosome compartments identified by TEM, these data are further confirmed. In detail, LF produces a slight decrease in LAMP1 within cytosol (Figure 16A) while it dramatically increases the number of LAMP1 positive vacuoles (Figure 16B) and a similar robust increase in the number of LAMP1 particles within vacuoles (Figure 16C). As a consequence, when the ratio of vacuolar vs. cytosolic LAMP1 was measured, LF alone dramatically increases LAMP1 polarization (Figure 16D). This scenario partly overlaps with what described for LC3. In fact, when the merging of LC3 with LAMP1 particles is shown (Figure 17A) and calculated (Figure 17B), LF produces over two-fold increase in the merging between LC3 and LAMP1, which strongly suggests the commitment of the enhanced reservoir of LC3 positive vacuoles, which is described following LF. In contrast, when METH alone was administered, immuno-fluorescence indicates an opposite trend of LAMP1 compared with what produced by LF alone (Figure 14), as representatively reported at TEM (Figure 15). The amount of cytosolic LAMP1 measured at ultrastructural stoichiometry following METH was not modified (Figure 16A), while the amount of LAMP1 positive vacuoles (Figure 16B) and the amount of LAMP1 particles within vacuoles were decreased (Figure 16C). This explains why the polarization of LAMP1 within vacuoles is dissipated (Figure 16D), which reduces the amount of LC3 + LAMP1 positive vacuoles (Figure 17). The pre-administration of LF restores these alterations produced by METH in the merging between autophagosome and lysosomes as well as the vacuolar compartmentalization of LAMP1.

This strongly indicates that LF per se is able to enhance autophagy by promoting the formation of autophagolysosomes. Moreover, LF is able to counteract the de-potentiation of autophagy induced by METH, by promoting the proper LC3 and LAMP1 vacuolar co-localization.

## 4. Discussion

The present study shows that LF is active directly on subcellular structures involved in METH-induced degeneration. In detail, LF significantly attenuates the amount of cell loss produced by METH; it counteracts the mitochondrial alterations induced by METH and mitigates the dissipation of autophagy-related proteins from the autophagy compartment, which is massively induced by METH. 

In detail, ultrastructural analysis of the specific autophagy proteins LC3 and LAMP1 reveals that METH, though increasing the number of vacuoles and LC3 immuno-gold particles, reduces the amount of LC3 or LC3 + LAMP1 positive vacuoles, thus de-potentiating autophagy. This effect is counteracted by LF. In fact, in cells pre-treated with LF, the number of LC3 positive vacuoles and the ratio between vacuolar and cytosolic LC3 immuno-gold particles, which was dissipated by METH, is re-established again. The prominently vacuolar localization of LC3 indicates that autophagy vacuoles are properly committed, and autophagy is ongoing. Remarkably, when LF is administered alone, it augments the number of vacuoles, the number of LC3 positive vacuoles, and the number of LC3 particles within vacuoles, as well as the ratio between vacuolar and cytosolic LC3 immuno-gold particles compared with controls. LF alone decreases the amount of cytosolic LC3. These findings are partially replicated by measuring the lysosomal marker LAMP1 at subcellular level. In fact, LF, in combination with METH, re-establishes the amount of LAMP1 positive vacuoles and the amount of LAMP1 particles within vacuoles, which were dissipated by METH. More interestingly, when co-localization of LC3 and LAMP1 are measured, a marked increase in LC3 and LAMP1 double-stained vacuoles is found in either LF alone or METH + LF-treated cells. All these effects tightened each other. In fact, the dissipation of specific proteins such as LC3 and LAMP1 from autophagy vacuoles, as well as the dissipation of these proteins along with Cathepsin D from autophagolysosome vacuoles, de-potentiates the autophagy machinery [87]. The loss of efficacy in the autophagy pathway leads to the enhancement of METH toxicity [46], while the potentiation of autophagy protects from METH toxicity [68]. This potentiation achieved by classic autophagy inducers such as rapamycin is mostly evident by a polarization of autophagy-related proteins within autophagy vacuoles. This phenomenon counteracts the dissipation of the very same proteins from autophagy vacuoles, which is induced by METH [68]. In fact, METH-induced cell loss is dependent on this chain of events. Similarly, the effects on the mitochondrial compartment evidenced here following LF administration is consistent with the effects produced by the autophagy inducer rapamycin [84]. In fact, just like rapamycin, LF increases the total amount of mitochondria by increasing selectively the number of heathy mitochondria. Additionally, such a mitochondrial effect counteracts the effect produced by METH, which increases mitochondria by augmenting damaged mitochondria. In fact, in the present study, the administration of LF restores the number of healthy mitochondria in METH-treated cells to one similar to that of controls. Removal of altered mitochondria by autophagy (i.e., mitophagy) and generation of new mitochondria (i.e., mitochondriogenesis) are synergistically connected [84,89,90]. This was recently shown following the autophagy inducer rapamycin, which also promotes the expression of specific genes committed in the biogenesis of mitochondria [84]. This parallelism suggests further experimental investigations following LF administration to analyze the molecular biology of its effects in promoting genes related to mitophagy, mitochondriogenesis, autophagy, and lysosomal degradation. These studies are planned in our labs to analyze the potential of neuronal plasticity induced by LF. These latter effects are further suggested by the phenotypic alterations, which were induced by LF concerning the number of branches and the expression of the neuro-filament proteins βIII-tubulin. These effects suggest that LF plays an important role in the maturation phenomena, both during development and regeneration within the CNS. In fact, LF dose- and time-dependently increases the length of cell branches and the number of cells with branches. When LF is administered at high doses, deleterious effects prevail; in fact, up to 16 μM, such a neuronal plasticity increases, and for higher doses, such an effect fades away. Similarly, cell viability is maintained by LF 16 μM, while it progressively decreases for high LF doses, such as 160 and 320 μM. This is consistent with previous data showing that LF induces cell loss at doses of 200 μg/mL corresponding roughly to 64 μM in PC12 cells [91]. The ability of LF to induce the neurite outgrowth was previously described in similar cultures of sympathetic neurons [92]. Similarly, LF-induced expression of βIII-tubulin neuro-filaments was demonstrated in neuroblastoma cells [93]. Our data confirms a very recent study showing that LF promotes neurite outgrowth in PC12 cells [94]. In fact, class III β-tubulin is a neuron-associated major component of microtubules [95]. In particular, its expression occurs very early within cells, which are committed to differentiate into neurons [96,97,98]. As part of the cytoskeleton, βIII-tubulin plays an essential role in determining cell shape [98,99]. In fact, the expression of βIII-tubulin is greatest during development of the CNS [100] for axon guidance and maturation [101]. Accordingly, LF-induced phenotype alterations towards a marked cell differentiation were shown in glioblastoma cells [102]. Previous studies analyzed the effects of LF at mitochondrial level. In fact, LF-induced mitochondrial preservation is seminal for LF-induced protection against 1-methyl-4-phenylpyridinium (MPP+)-induced degeneration of midbrain DA neurons [34] and administration of the scrapie-like fragment of the prion protein [103]. This is in line with findings showing that LF preserves the integrity of mitochondria even at a systemic level. LF was shown to reduce mitochondrial damage even within liver cells [104,105], and it improves mitochondrial morphology and dynamics within myocardium from aged mice [42]. It is likely that such a strong effect on mitochondria produced by LF may cover other mechanisms beyond mitophagy and mitochondriogenesis. In fact, LF promotes mitochondrial calcium homeostasis via modulation of the phosphoinositide 3-kinase/AKT-dependent signaling pathway [34]. In addition, LF directly prevents mitochondrial dysfunction through a marked antioxidant and ROS scavenging activity [103,104]. These effects may further explain data produced in the present study, where LF increases the number of mitochondria, and counteracts mitochondrial alterations induced by METH. These additional effects of LF need to be considered along with mitophagy and mitochondriogenesis, which are likely to play a major role since mitochondrial integrity is strictly dependent on the integrity of the autophagy pathway [89,106]. In fact, mitochondria dysfunction occurs in cells where autophagy is defective, while autophagy activation, apart from promoting removal of dysfunctional mitochondria, triggers increased mitochondriogenesis and mitochondria plasticity, which increase the amount of functional and well-shaped mitochondria [84]. Impairment of autophagy plays a pivotal role in METH-induced neurotoxicity [66], and autophagy stimulation is protective against cell alterations and cell death produced by METH exposure [46,48,68].

The present study demonstrates that LF enhances autophagy by increasing autophagy and lysosomal compartmentalization of committed proteins, which instead dissipate from vacuolar compartments following METH. This engagement of autophagy proteins within specific compartments leads to an effective co-localization of active autophagy proteins such as LC3, and lysosomal markers Cathepsin D or the autophagy/lysosomal protein LAMP1. Ultrastructural investigations indicate vacuolar re-allocation of LC3 and LAMP1 particles by LF to counteract METH-induced dissipation from autophagy vacuoles. 

The specificity of LF as neuroprotectant relates to the specific cell phenotype. In fact, the induction of autophagy is key to counteract METH toxicity, which does not occur in non-DA-containing cell lines. In this study, we could not detect METH toxicity in U87MG cells. In this cell line, LF stimulates autophagy and the neurofilament protein βIII-tubulin. However, in U87MG cells, these effects promote cell differentiation rather than neuroprotection. Such a phenotype-dependent variability in the effects of LF confirms what has already been published in experimental GBM, where LF was shown to possess anti-proliferative and differentiating properties [102]. Thus, LF promotes ubiquitous biochemical pathways, which possess a different relevance depending on specific cell phenotype (i.e., neuroprotective or cytostatic effects).

## 5. Conclusions

The present model, apart from eliciting a novel compound to protect against METH toxicity, addresses a beneficial effect of LF directly on mitochondria and the autophagy status. LF is widely distributed in the brain, being abundant within the substantia nigra [20], a variety of brainstem nuclei and the olfactory systems [21]. LF quickly crosses the blood–brain barrier (BBB) via receptor-mediated transcytosis and accumulates within brain endothelial cells [107]. In fact, LF neuroprotection is also related to stimulation of glial and endothelial cells to produce growth factors [36]. In line with this, LF receptors occur widely in the brain, within neurons (both cell body and axons), cerebral vessels, and glia [29,108]. 

In particular, LRP are prominently involved [93,109,110]. In fact, LRP expressed on the surface of the brain endothelial cells allow LF to cross the BBB [90]. It is remarkable that PC12 cells used in the present study do express LRP [111].

LF levels change during neurodegenerative disorders [20,24,25,26]. In fact, LF accumulates in the pathological hallmarks of dementia and Parkinsonism. Thus, LF is present within amyloid-beta deposits in the brain of AD patients [20], as well as in the mesencephalon of PD patients [26] and PD mouse models [28,112].

These findings call for an active involvement of LF in the molecular mechanisms underlying neurodegeneration. In detail, if accumulation of LF in the brain of PD and AD patients may be the consequence of dysregulated iron homeostasis [112,113,114,115], an alternative hypothesis should be considered as well. In fact, LF may accumulate as an attempt to compensate for neurodegeneration, thus playing a protective role to preserve neurons [34]. This alternative hypothesis is consistent with data provided in the present and previous studies. In fact, LF protects DA neurons against METH-induced neurodegeneration (present study) and that induced by the parkinsonian neurotoxin MPTP [36,37,38]. 

## Data Availability

The data used to support the findings of this study are available from the corresponding author upon request.

## Supplementary Materials

The following are available online at www.mdpi.com/article/10.3390/nu13103356/s1 Figure S1. Time-course of a non-toxic dose of LF on PC12 cells, Figure S2. Time-course of a frankly toxic dose of LF on PC12 cells, Figure S3. Dose-response of LF on FJB staining in U87MG cells, Figure S4. Dose-response of LF on H&E staining, Figure S5. METH has no toxic effects on U87MG cells, Figure S6. METH does not alter FJB-responsiveness in U87MG cells, Figure S7. LF enhances the growth of cell elongations in U87MG cells, Figure S8. Representative III-tubulin immuno-fluorescence, Figure S9. III-Tubulin western blotting, Figure S10. LF induces LC3 compartmentalization within vacuoles in U87MG cells.
