# Supplementary material for: Lactoferrin Protects against Methamphetamine Toxicity by Modulating Autophagy and Mitochondrial Status
Source: Nutrients. 2021 Sep 25;13(10):3356. doi: 10.3390/nu13103356 (PMC8537867; doi:10.3390/nu13103356)
Supplement: Supplementary file 1 [file nutrients-13-03356-s001.zip › nutrients-1357826-supplementary.pdf]

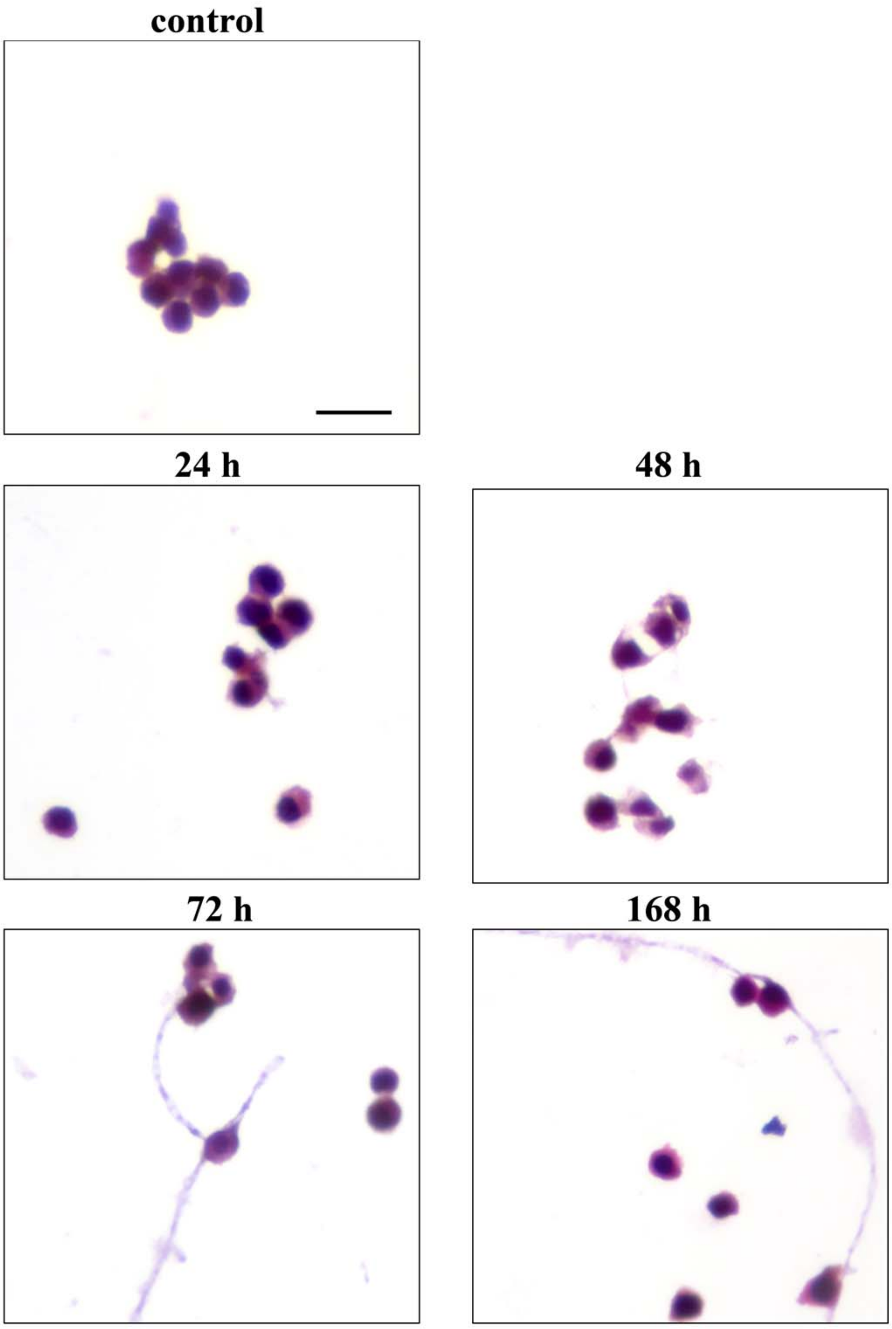

Supplementary Figure S1. Time-course of a non-toxic dose of LF on PC12 cells. Representative pictures of H&E-stained PC12 cells related to a non-toxic dose (16 μM) of LF. Scale bar = 27 μm.

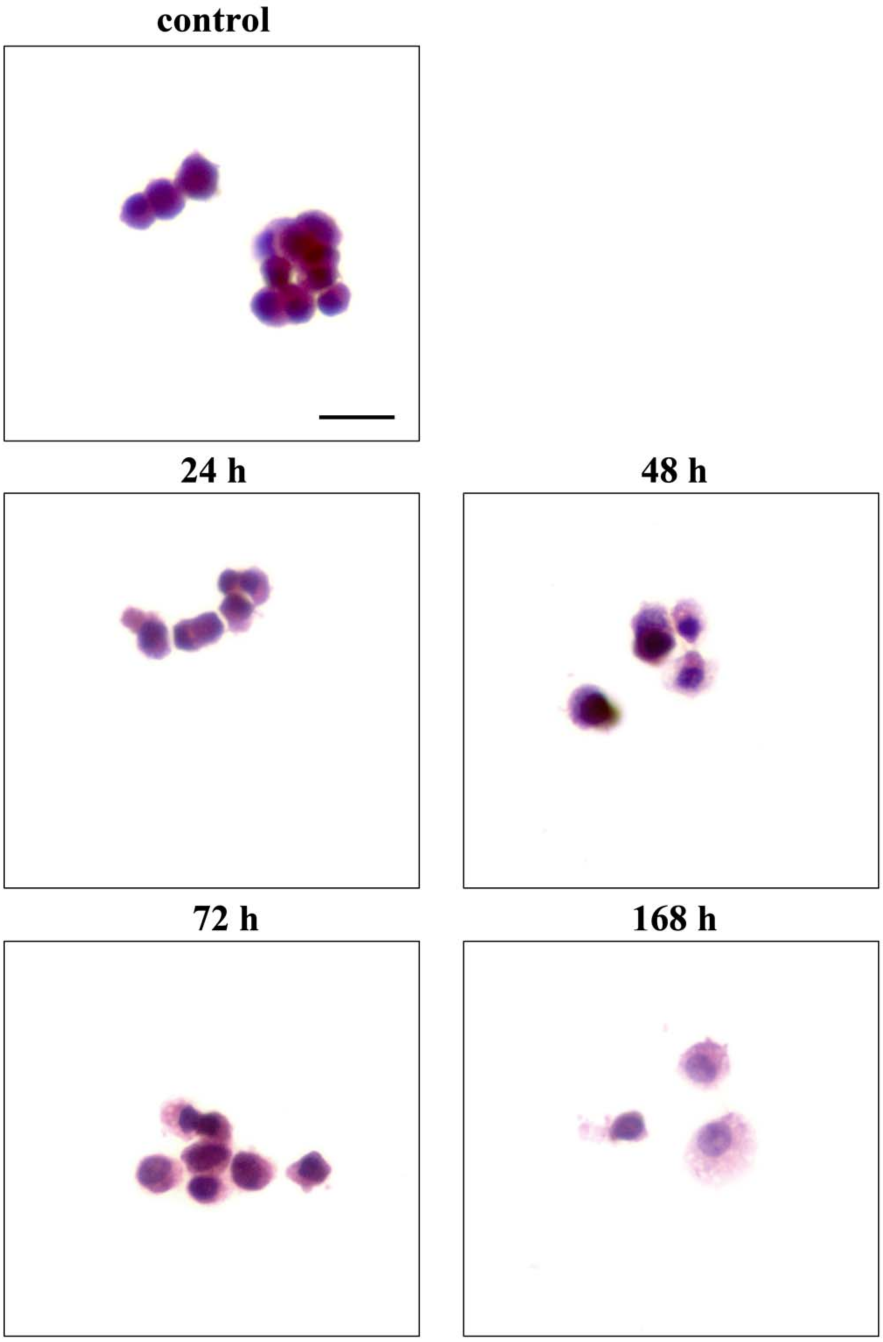

Supplementary Figure S2. Time-course of a frankly toxic dose of LF on PC12 cells. Representative pictures of H&E-stained PC12 cells related to a frankly toxic dose (320 μM) of LF. Scale bar = 27 μm.

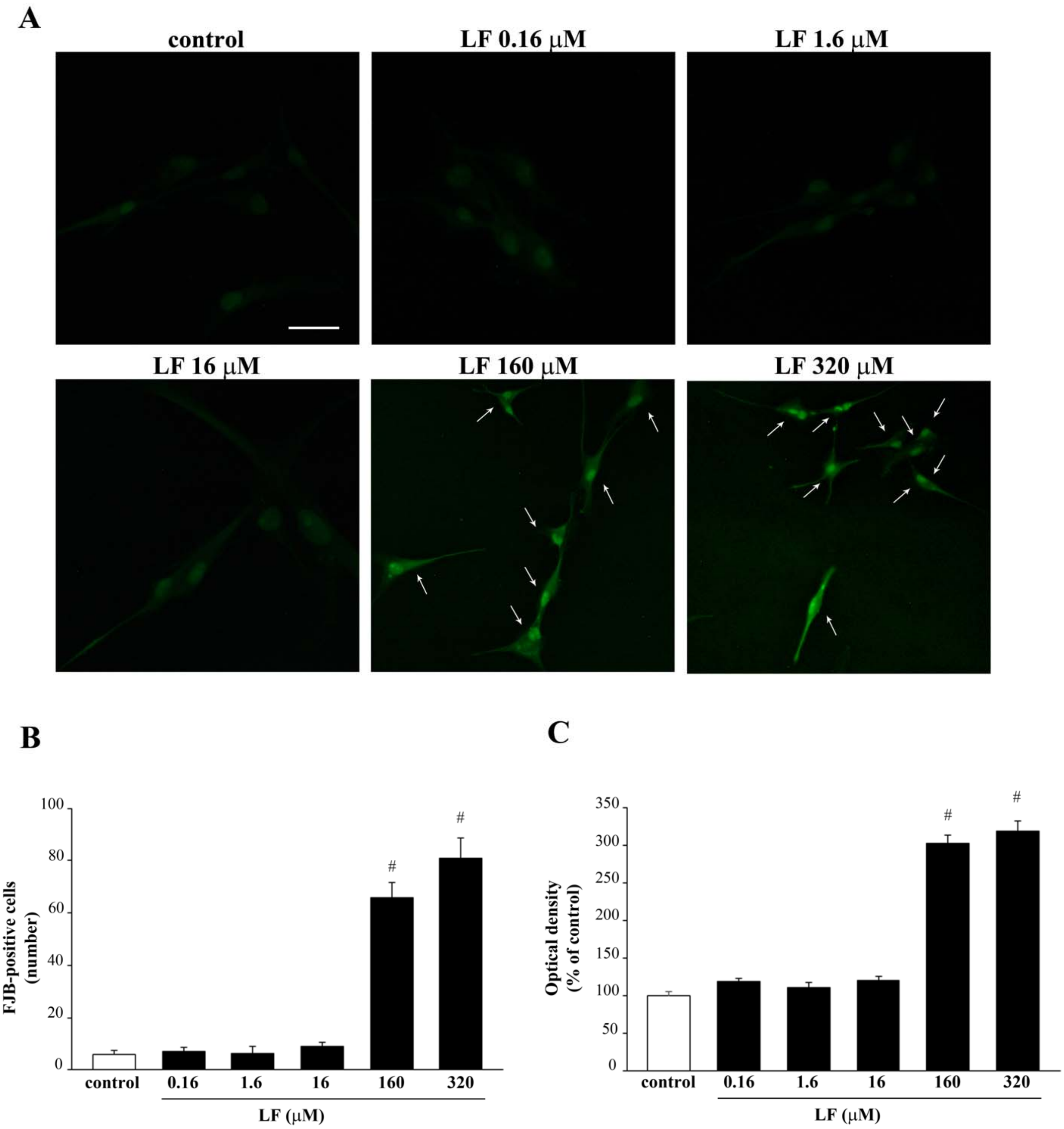

Supplementary Figure S3. Dose-response of LF on FJB staining in U87MG cells. (**A**) Representative pictures of FJB-stained U87MG cells after treatment with various doses (from 0.16 μM up to 320 μM) of LF for 72 hours. The number and intensity of FJB fluorescent cells is reported in graphs (**B**) and (**C**), respectively. Arrows indicate FJB intensely positive cells. #P≤0.05 compared with control and LF 0.16 μM, 1.6 μM and 16 μM. Scale bar = 26 μm.

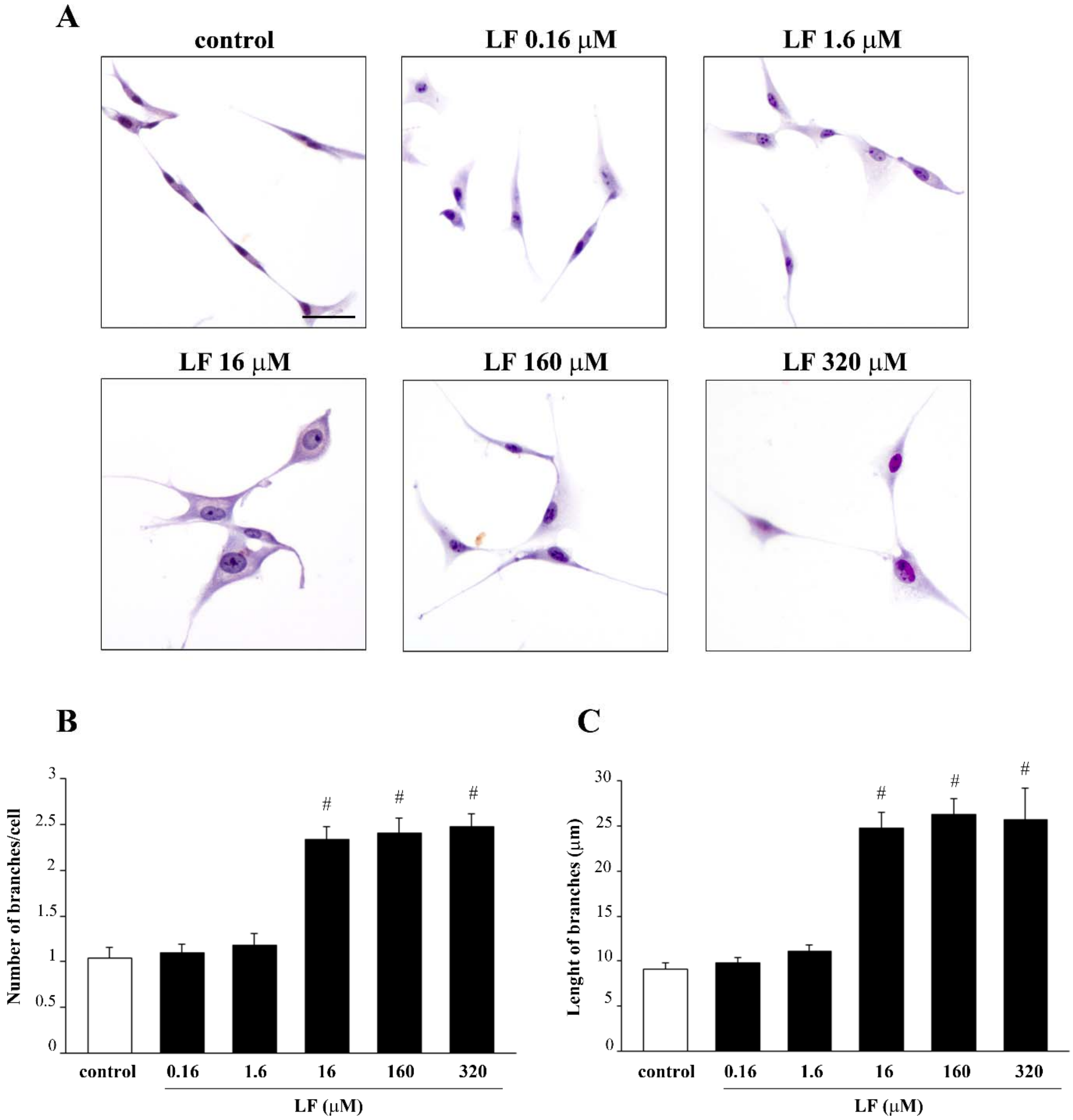

Supplementary Figure S4. Dose-response of LF on H&E staining. (A) Representative pictures of H&E-stained U87MG cells after treatment with various doses (from 0.16 μM up to 320 μM) of LF for 72 hours. Graphs report the number (**B**) and the length (**C**) of cell branches induced by various doses of LF. #P<0.05 compared with control, LF 0.16 μM and LF 1.6 μM. Scale bar = 24 μm.

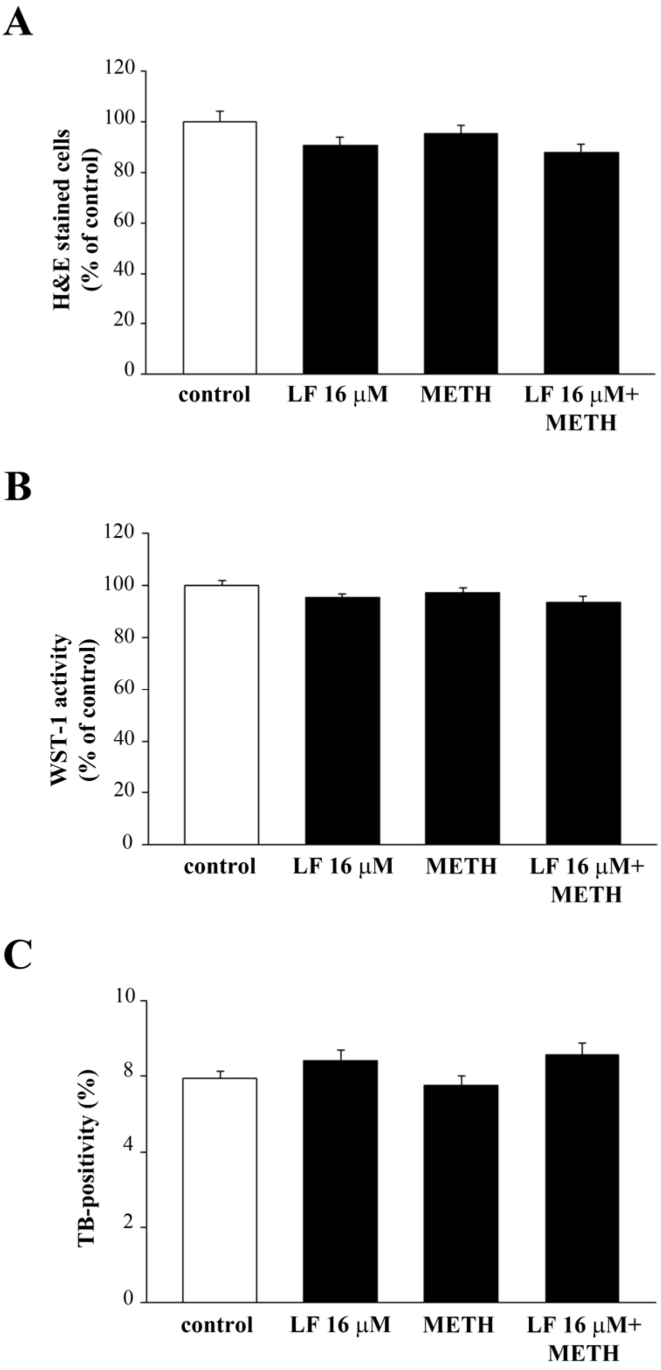

Supplementary Figure S5. METH has no toxic effects on U87MG cells. Assessment of U87MG cells viability through (**A**) H&E staining, (**B**) WST-1 assay, and (**C**) TB staining following administration of METH, alone or in combination with LF at 16 μM for 72 hours.

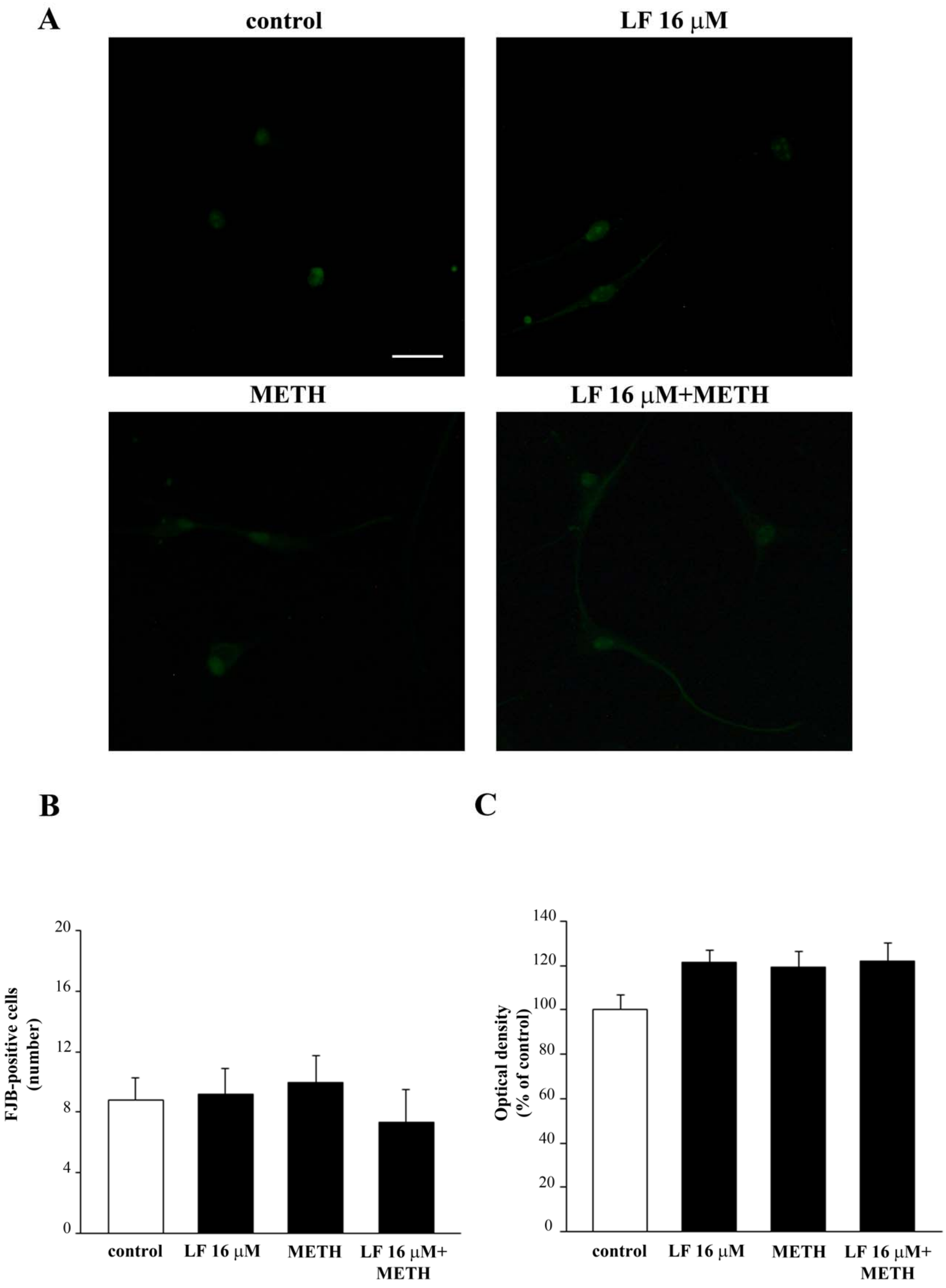

Supplementary Figure S6. METH does not alter FJB-responsiveness in U87MG cells. Representative pictures of FJB-stained U87MG cells after combined LF and METH treatment. The graphs (**B**, **C**) report the number and the intensity of FJB fluorescent cells, respectively. Scale bar = 26 μm.

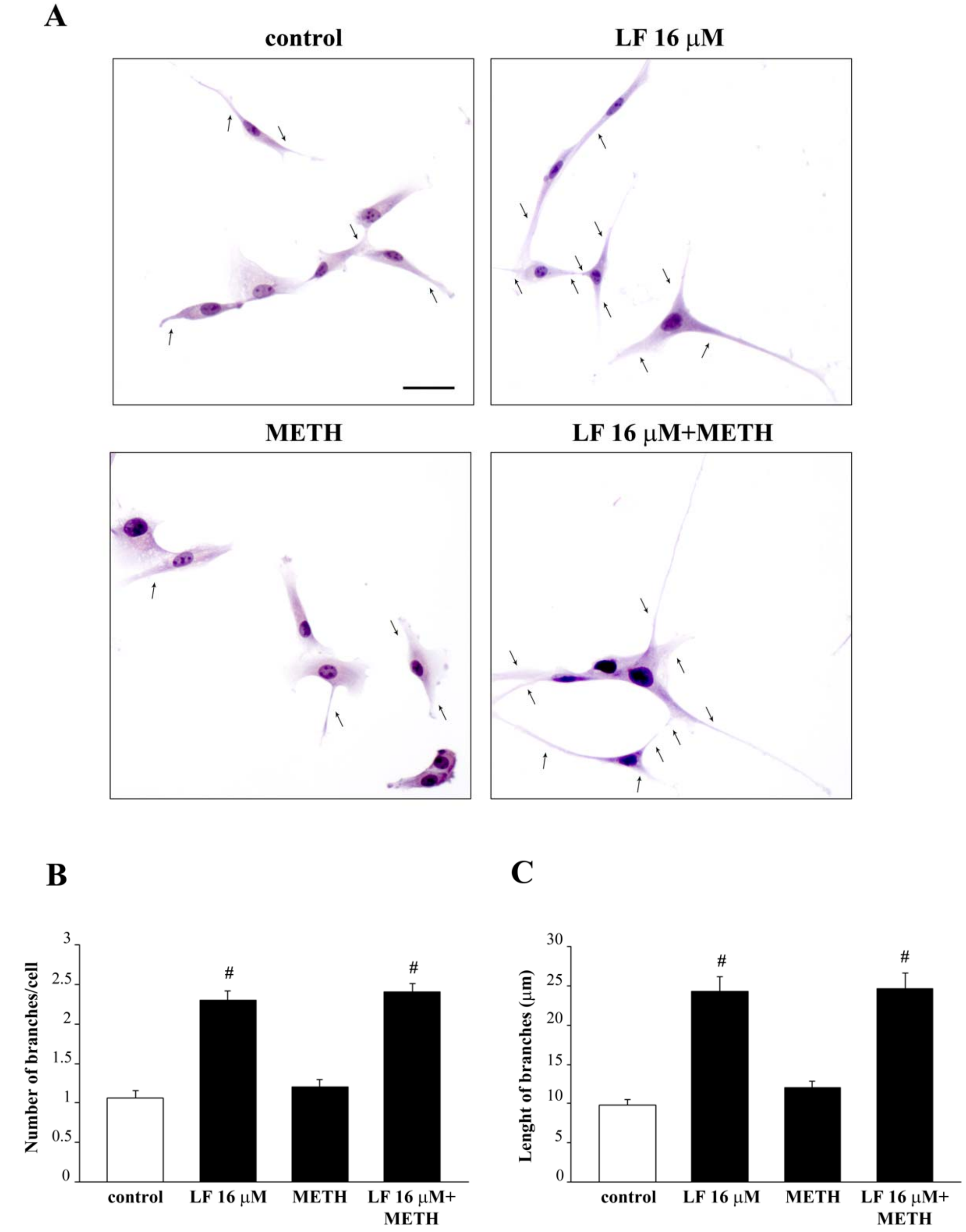

Supplementary Figure S7. LF enhances the growth of cell elongations in U87MG cells. Representative pictures of H&E-stained branching out of U87MG cells after combined LF and METH treatment. Arrows indicate cell elongations. The histograms report (**B**) the mean number of branches per cells and (**C**) the mean length of branches observed in each experimental group. #P≤0.05 compared with control and METH. Scale bar = 26 μm.

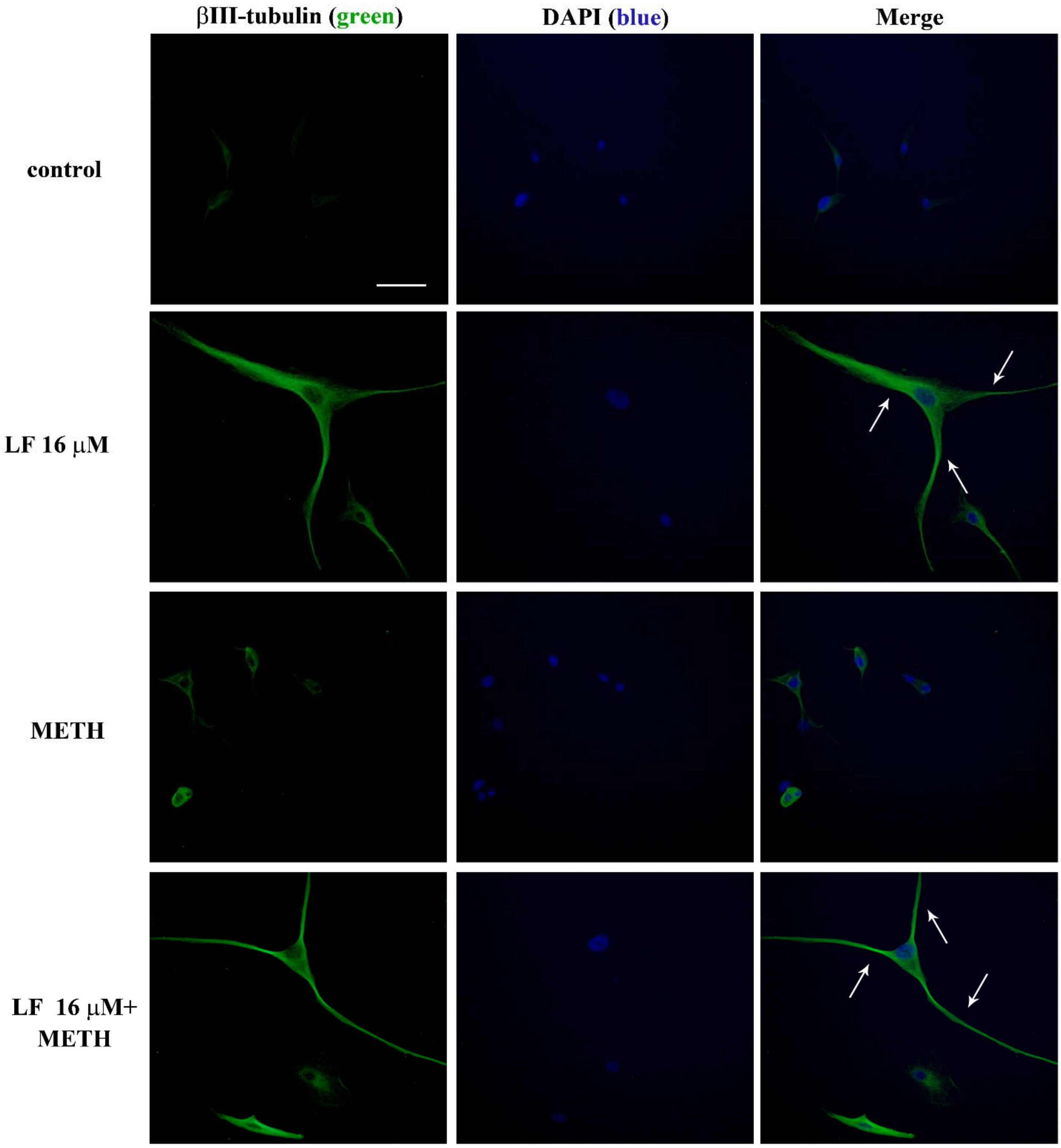

Supplementary Figure S8. Representative βIII-tubulin immuno-fluorescence. Representative pictures of βIII-tubulin immuno-fluorescence after single and combined LF+METH administration. Arrows indicate βIII-tubulin immuno-fluorescent cell branches. Scale bar = 30 μm.

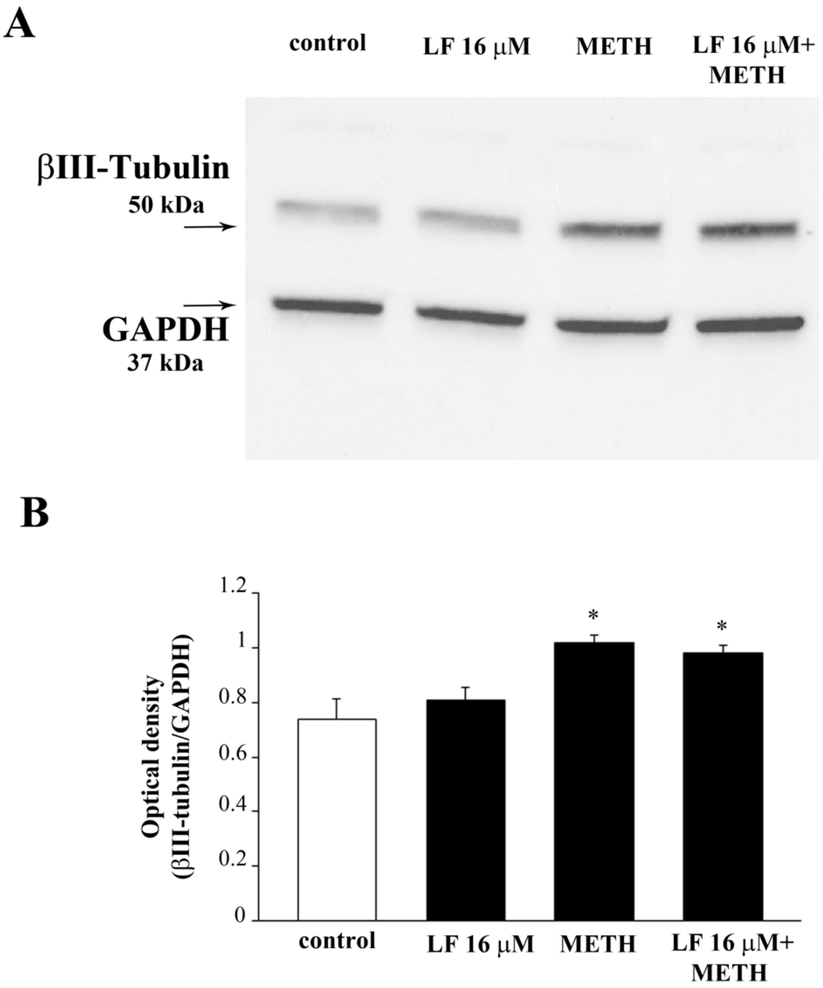

Supplementary Figure S9. βIII-Tubulin western blotting. Representative western blotting for the neuro-filament marker βIII-tubulin in U87MG control cells and in cells treated with LF and METH, alone or in combination is shown. (**B**) The graph reports the ratio between the optical density of βIII-tubulin and the housekeeping protein GAPDH. *$P<0.05$ compared with control.

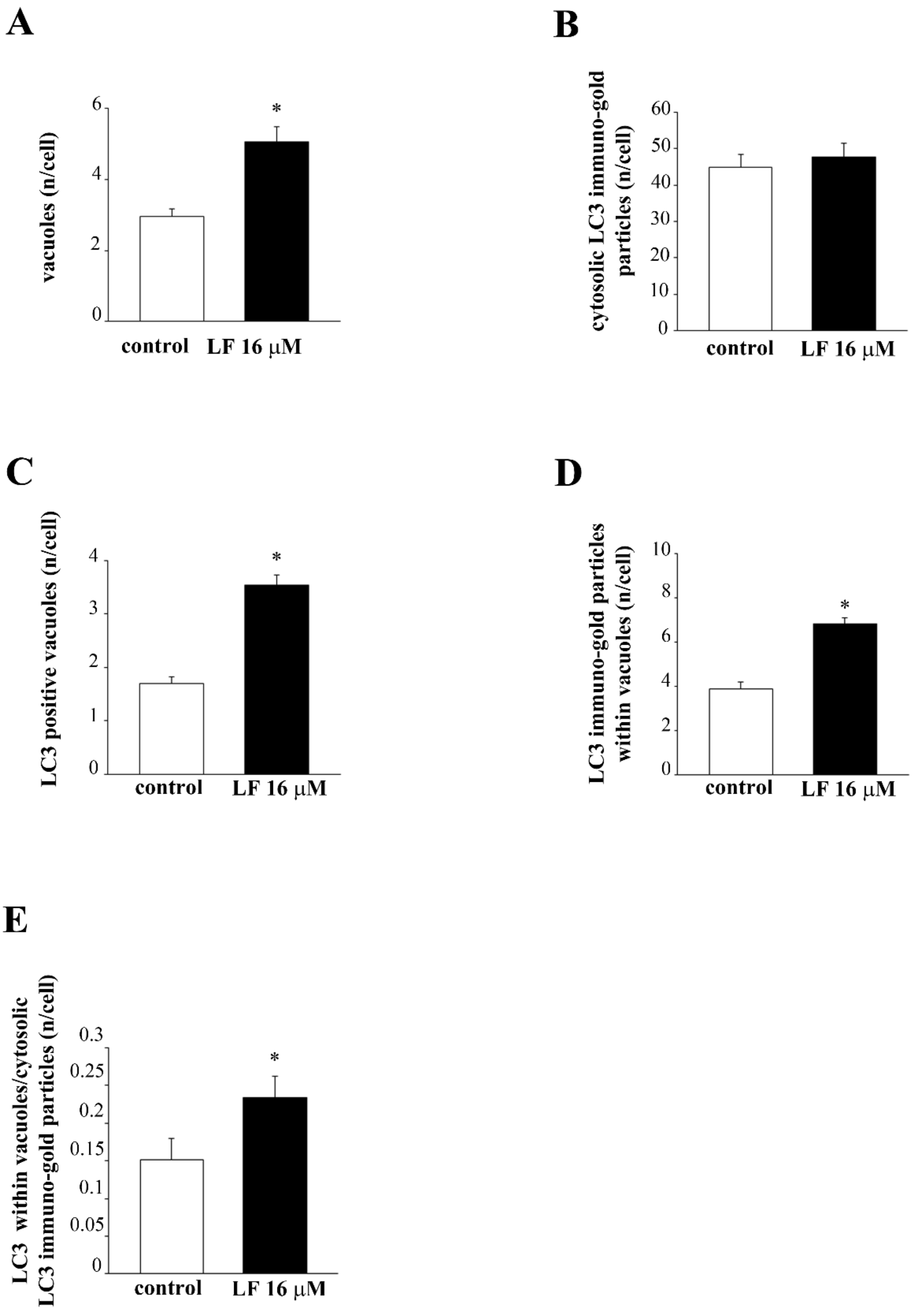

Supplementary Figure S10. LF induces LC3 compartmentalization within vacuoles in U87MG cells. Quantitative ultrastructural morphometry for LC3 is reported in the graphs (**A-E**). *P≤0.05 compared with control.
